# Human Amniotic Fluid Mesenchymal Stem Cells from Second- and Third-Trimester Amniocentesis: Differentiation Potential, Molecular Signature, and Proteome Analysis

**DOI:** 10.1155/2015/319238

**Published:** 2015-08-17

**Authors:** Jurate Savickiene, Grazina Treigyte, Sandra Baronaite, Giedre Valiuliene, Algirdas Kaupinis, Mindaugas Valius, Audrone Arlauskiene, Ruta Navakauskiene

**Affiliations:** ^1^Department of Molecular Cell Biology, Vilnius University, LT-08662 Vilnius, Lithuania; ^2^Proteomic Center, Institute of Biochemistry, Vilnius University, LT-08662 Vilnius, Lithuania; ^3^Clinics of Obstetrics and Gynecology, Faculty of Medicine, Vilnius University, M. K. Čiurlionio Gatvė 21, LT-03101 Vilnius, Lithuania

## Abstract

Human amniotic fluid stem cells have become an attractive stem cell source for potential applications in regenerative medicine and tissue engineering. The aim of this study was to characterize amniotic fluid-derived mesenchymal stem cells (AF-MSCs) from second- and third-trimester of gestation. Using two-stage protocol, MSCs were successfully cultured and exhibited typical stem cell morphological, specific cell surface, and pluripotency markers characteristics. AF-MSCs differentiated into adipocytes, osteocytes, chondrocytes, myocytes, and neuronal cells, as determined by morphological changes, cell staining, and RT-qPCR showing the tissue-specific gene presence for differentiated cell lineages. Using SYNAPT G2 High Definition Mass Spectrometry technique approach, we performed for the first time the comparative proteomic analysis between undifferentiated AF-MSCs from late trimester of gestation and differentiated into myogenic, adipogenic, osteogenic, and neurogenic lineages. The analysis of the functional and expression patterns of 250 high abundance proteins selected from more than 1400 demonstrated the similar proteome of cultured and differentiated AF-MSCs but the unique changes in their expression profile during cell differentiation that may help the identification of key markers in differentiated cells. Our results provide evidence that human amniotic fluid of second- and third-trimester contains stem cells with multilineage potential and may be attractive source for clinical applications.

## 1. Introduction

Human amniotic fluid (AF) collected during amniocentesis between 15th week and 19th week of gestation is used for the routine prenatal diagnosis of wide range of fetal abnormalities and genetic diseases [1–4]. AF represents a heterogeneous cell population derived from placental membranes, fetal skin, and digestive, respiratory, and urinary tract. AF from amniocentesis samples contains terminally differentiated cells with limited proliferation capacity and fetal mesenchymal stem cells with multilineage differentiation potential [5, 6]. Recently, AF was considered as an attractive source of stem cells of mesenchymal origin for therapeutic applications and with low risk of tumorigenicity [7]. Multiple approaches have been used to isolate and characterize these stem cell types. Based on morphological characteristics, AF colonies consist of adherent “spindle”-shaped fibroblast-like cells and “round”-shaped epithelioid cells [8] but epithelial cells disappear during propagation of mixed primary cell cultures. To date, distinct clonal populations were isolated from AF by dilution and direct plating, including phenotypically and functionally distinct stromal cell clones, long-lived epithelial cells, and senescent population [9]. Clonal populations were established with cloning rings or mechanically picked up, with immunoselection of cells expressing the receptor for Steel factor (C-kit+) or magnetic cell sorting for CD117+ [10–12]. The majority of isolated AFSCs shared a multipotent mesenchymal phenotype and exhibited high proliferation and differentiation potential [5, 13, 14]. AF-MSCs differentiation into adipocytes, chondrocytes, osteocytes, cardiomyocytes, and neuronal cells have been reported* in vitro* and* in vivo* [15–19].

Cell populations in amniotic fluid have great diversity and variation among amniocentesis samples from different donors, time of gestation, and cultivation. So far, the second-trimester amniocentesis samples are usually used for research work, but at this gestational time it is impossible to collect a larger volume of amniotic fluid and increased risk of uterine contamination and miscarriage. To date, little is known about the biological characteristics of the third-trimester AF-MSC, which may serve as a rich source of stem cells for autologous therapy [20, 21]. These potential advantages led to the comparative investigation of AF-MSCs from late second- and third-trimester. In this study, we demonstrated that AF-MSCs can be successfully isolated and expanded from both second- and third-trimester amniotic fluids, which maintain the expression of multipotency markers and are inducible to different cell lineages. Proteome analysis documented the similarities and specific changes in the expression profiles of undifferentiated AF-MSCs and differentiated into myogenic, adipogenic, osteogenic, and neurogenic lineages.

## 2. Materials and Methods

### 2.1. Isolation and Expansion of Mesenchymal Stem Cells from Amniotic Fluid

Samples (about three to five milliliters) were obtained by biopsy (amniocentesis) from mid second- (16–24 weeks, *n* = 6) or third-trimester (28–34 weeks, *n* = 3) amniotic fluid from healthy woman who needed prenatal diagnosis but no abnormalities were revealed by genetic analysis. Samples were maintained at room temperature for about 4 hours prior to isolation of amniotic cells using two-stage protocol [22]. The sample was centrifuged at 1,800 rpm for 20 min, the supernatant was removed, and the cell pellet was washed once in DMEM medium (Sigma-Aldrich Ltd.) without serum to remove blood and cell debris. After centrifugation, the cell pellet was resuspended in 5 mL of growth medium AmnioMAX-C100 basal with AmnioMAX-C100 supplement (Gibco, Life Technologies, Grand Island, NY, USA), 100 U/mL penicillin, and 100 *μ*g/mL streptomycin (Gibco, Grand Island, NY, USA) and plated in a 25 cm^2^ culture flask (TPP, Switzerland). Amniocytes were incubated for 10–15 days at 37°C in 5% CO_2_, when first colonies appeared (first stage). For culturing AF-MSCs (second stage), nonadhering AF cells were collected from primary culture and further expanded in a new 25 cm^2^ culture flask at 37°C in 5% CO_2_. After the appearance of cell colonies, the growing medium was changed every 3 days. Cells were subcultured into higher passages at approximately 80% confluence with 0.05% trypsin-EDTA (Gibco, Life Technologies, Grand Island, NY, USA), A morphologically homogeneous population of fibroblast-like cells was obtained after two rounds of subculture.

### 2.2. Flow Cytometry Analysis

For identification of the phenotype of AF-MSCs from passages 4-5, cells were collected by centrifugation at 1,200 rpm for 6 min, washed once in phosphate buffered saline (PBS) with 0.2% fetal calf serum (FCS), and centrifuged again. A total of 5 × 10^5^ cells were resuspended in 50 *μ*L of PBS with 1% BSA and incubated with fluorescein isothiocyanate- (FITC-) conjugated mouse anti-human antibodies against CD44 (Invitrogen), CD34 (Miltenyi Biotech), CD90 (Molecular Probes, Life technologies) or phycoerythrin- (PE-) labelled CD105 (Invitrogen), and appropriate isotype control mouse IgG2A-FITC (Miltenyi Biotec) or IgG1-PE (Molecular Probes, Life Technologies). Samples were incubated in the dark at 4°C for 30 min and analysis was performed on a flow cytometer BD FACSCanto II (Beckton and Dickinson) with BD FACSDiva software.

### 2.3. Differentiation Assays

AF-MSCs differentiation capacity was performed in a monolayer as detailed by manufacturer's protocol using commercially available STEMPro Differentiation kits (Gibco, Invitrogen cell culture). AF-MSC were cultured at 80%–90% confluence and subsequently differentiated with STEMPro Differentiation medium at 37°C in 5% CO_2_. For cell staining, AF-MSCs were seeded into a 4-well (3.85 cm^2^) plate (Nunc, Thermo Scientific, Roskilde, Denmark) at a 1 × 10^4^ cells/cm^2^. Each cell population was differentiated in 3 replicates using undifferentiated cells for controls. During cell differentiation, the medium was replaced every 2-3 days. For gene expression studies, AF-MSCs were cultured in a T-25 flask.

For adipogenic differentiation, AF-MSCs were cultured at 80% confluence and subsequently differentiated with STEMPro Adipogenic Differentiation medium for 14–18 days at 37°C in 5% CO_2_. After specific period of cultivation, formation of intracellular lipid droplets was monitored by Oil Red O staining. In brief, cells were washed with 60% isopropanol, dried, and stained for 10 min in Oil Red O solution freshly diluted into distilled water at a ratio of 3 : 2, followed by three washes in distilled water.* Adiponectin* expression was determined by RT-qPCR.

For osteogenic differentiation, AF-MSCs after seeding at a 1 × 10^4^ cells/cm^2^ into a 4-well plate or T-25 flask were cultivated in STEMPro Osteogenic Differentiation medium for 14–18 days at 37°C in 5% CO_2_, according to the manufacturer's instructions. Osteogenic differentiation was determined by Alizarin Red staining of the calcified extracellular matrix deposition. In brief, samples were fixed with 4% formaldehyde for 15 min at room temperature, washed twice with PBS, pH 4.2, and stained with 2% Alizarin Red in deionized water for 20 min at 37°C, followed by two washes in PBS.* Osteopontin* expression was determined by RT-qPCR.

For chondrogenic differentiation, micromass cultures were generated by seeding 20 *μ*L droplets of cells (1.6 × 10^4^ cells/microliter in growing medium) into individual wells of 4-well plate. Cells were allowed to attach for 4 h at 37°C in 5% CO_2_ under high humidity conditions before adding STEMPro Chondrogenic Differentiation medium for 14–18 days. Chondrogenic pellets were determined by staining for 30 min with 1% Alcian Blue in 3% acetic acid, followed by three washes in 3% acetic acid and finally in water.

For myogenic differentiation, cells were plated at a 1 × 10^4^ cells/cm^2^ and cultured at 80% confluence and then washed with PBS before the incubation in DMEM containing antibiotics and 2% of horse serum (Invitrogen) for 14–18 days with low serum media changes every 2-3 days. Multinucleated cells were visualized by phase contrast microscope (Nicon Eclipse TS100) after staining with 0.1% crystal violet in 20% ethanol, followed by washing in water.* Myogenin* expression was determined by RT-qPCR.

For neural differentiation, the induction medium containing 1 mM all-*trans*-retinoic acid (Sigma) in DMEM/F12 with GlutaMax and N2 supplement (Gibco, Life Technologies, Grand Island, NY, USA) was used after culturing cells at 60% confluence. A morphologic change to neuron-like cells with axonal outgrowth was visualised by phase contrast microscope after staining with 0.1% crystal violet.* Nestin* expression was determined by RT-qPCR.

### 2.4. RNA Isolation and RT-qPCR

The AllPrep RNA/Protein Kit (Qiagen) was used to isolate and purify total RNA and native proteins (subsequently used for proteomic analysis) simultaneously from a single sample of control or differentiated AF-MSCs. The mRNA, eluted from the AllPrep spin columns, was reverse transcribed to cDNA by Maxima First Strand cDNA Synthesis Kit (Thermo Scientific, Vilnius). For RT-qPCR, Maxima SYBR Green qPCR Master Mix (Thermo Scientific, Vilnius) on the Rotor-Gene 6000 system (Corbett Life Science) was applied. The relative gene expression was calculated by a comparative threshold cycle delta-delta Ct method. A comparative threshold cycle (Ct) was used to determine gene expression relative to GAPDH. Relative mRNA levels are reported as an* n*-fold difference of untreated cells. Student's *t*-test was used to perform statistical analysis and values of <0.05 were stated as statistically significant.

Forward (F) and reverse (R) primers (5′-3′) used in RT-qPCR are as follows:

 OCT-4 - F: CTCCTGGAGGGCCAGGAATC; R: CCACATCGGCCTGTGTATAT
 Nanog - F: CCTATGCCTGTGATTTGTGG; R: CCGGGACCTTGTCTTCCTTT
 Sox-2 - F: GGCAGCTACAGCATGATGCAGGAC; R: CTGGTCATGGAGTTGTACTGCAGT
 Osteopontin - F: GTCCAGTCTTACCTCTCAAACCT; R: ATGTGGTCAGCCAGCTCGTC
 Adiponectin - F: GGAGACAGCTACTCCCCAAGAT; R: GTCCAGTCTTACCTCTCAAACCT
 Nestin - F: CAGCTGGCGCACCTCAAGATG; R: AGGGAAGTTGGGCTCAGGACTGG
 Myogenin - F: CAGCGAATGCAGCTCTCCACA; R: AGTTGGGCATGGTTCATCTG
 GAPDH - F: AACTCTGGTAAAGTGGATATTG; R: GGTGGAATCATATTGGAACA

### 2.5. Filter-Aided Protein Sample Preparation (FASP) for Mass Spectrometry Analysis

Samples were concentrated on Amicon ultra-0.5 mL 30 kDa centrifugal filter unit and were denatured in 8 M urea, 100 mM DTT solution with continuous rotation at 800 rpm in the temperature controlled shaker for 3 hours at 37°C.

Trypsin digestion was done according to a modified FASP protocol as described by Wiśniewski et al. [23]. Briefly, samples were washed with buffer containing 8 M urea. The proteins were alkylated using iodoacetamide. Buffer was exchanged by washing two times with 50 mM NH_4_HCO_3_ and proteins digested overnight with TPCK Trypsin 20233 (Thermo Scientific, USA). After overnight digestion, peptides were recovered by centrifugation and then two additional washes using 50% CH_3_CN were combined, acidified, lyophilized, redissolved in 0.1% formic acid, and then analysed by mass spectrometry.

### 2.6. Liquid Chromatography and Mass Spectrometry

Peptides were loaded on reversed-phase trap column PST C18, 100 Å, 5 *μ*m, 180 *μ*m × 20 mm (Waters Corporation, UK) with a flow rate of 15 *μ*L/min using loading buffer of 0.1% formic acid and subsequently separated on HSS-T3 C18 1.8 *μ*m, 75 *μ*m × 250 mm analytical column (Waters Corporation, UK) in 90 min linear gradient (A: 0.1% formic acid, B: 100% CH_3_CN, and 0.1% formic acid) at a flow rate of 300 nl per min.

The nano-LC was coupled with HDMS Synapt G2 mass spectrometer (Waters Corporation, UK). Data were acquired using Masslynx version 4.1 software (Waters Corporation, UK) in positive ion mode. LC-MS data were collected using data independent acquisition (DIA) mode MSE in combination with online ion mobility separation. The trap collision energy of mass spectrometer was ramped from 18 to 40 eV for high-energy scans in MSE mode. The trap and transfer collision energy for high-energy scans in HDMS mode was ramped from 4 to 5 eV and from 27 to 50 eV. For both analyses, the mass range was set to 50–2,000 Da with a scan time set to 0.9 seconds. A reference compound [Glu1]-fibrinopeptide B (Waters Corporation, UK) was infused continuously (500 fmol/*μ*L at flow rate 500 nL per min) and scanned every 1 minute for online mass spectrometer calibration purpose. The samples were run in triplicate.

### 2.7. Data Processing, Searching, and Analysis

Raw data files were processed and searched using ProteinLynx Global SERVER (PLGS) (Waters Corporation, UK). The following parameters were used to generate peak lists: (i) minimum intensity for precursors was set to 100 counts, (ii) minimum intensity for fragment ions was set to 30 counts, and (iii) intensity was set to 500 counts. Processed data was analysed using trypsin as the cleavage protease, one missed cleavage was allowed, fixed modification was set to carbamidomethylation of cysteines, and variable modification was set to oxidation of methionine. Minimum identification criteria included 2 fragment ions per peptide, 5 fragment ions per protein, and minimum of 2 peptides per protein. The false discovery rate (FDR) for peptide and protein identification was 4%. The identified proteins were analysed for their functional properties by using UniprotKB/SwissProt human database (2015-01-29). The bioinformatics pipeline ISOQuant (http://www.immunologie.uni-mainz.de/isoquant/) was used for label-free quantification. *t*-tests were performed on data to evaluate the difference between groups (MarkerView software, AB Sciex).

## 3. Results

### 3.1. Characterization of MSC Population from Amniotic Fluid of Late Second- and Third-Trimester

**Figure 1:**
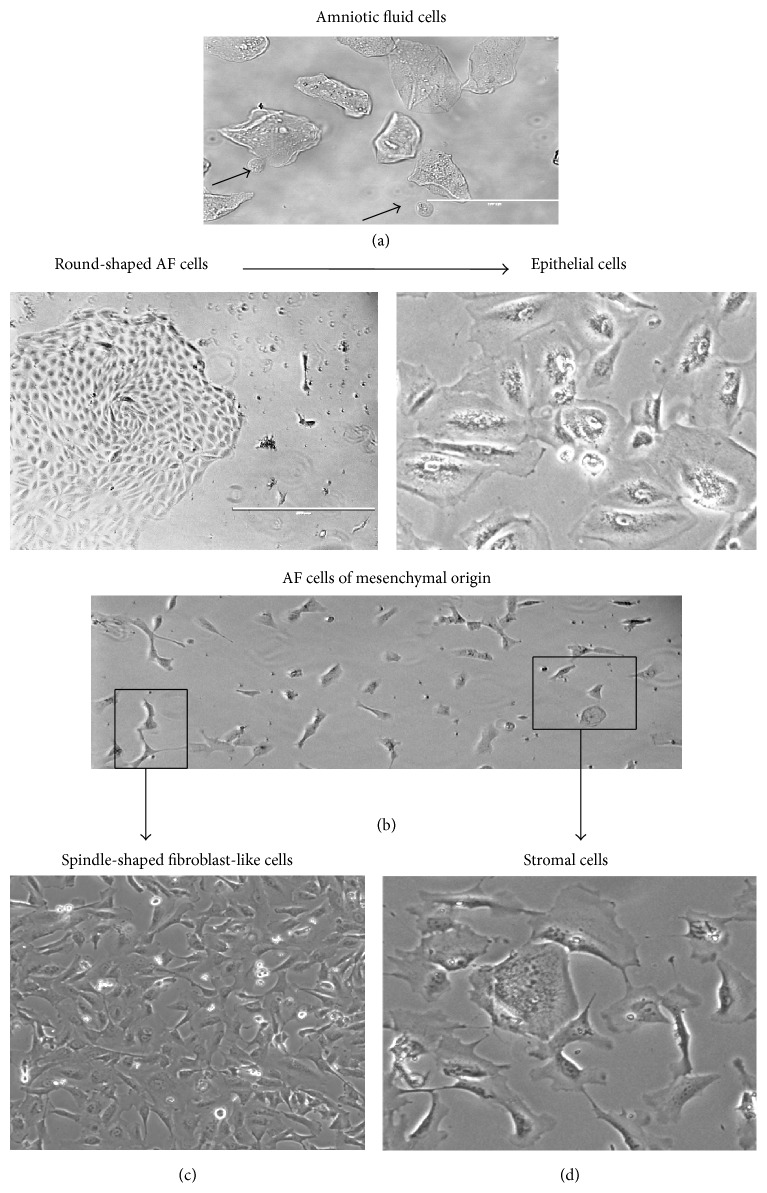
Morphological characteristics of AF cells. (a) Amniotic fluid cells from amniocentesis sample. (b) The colony appearance of epithelial type at 10–15 days after initiation of the primary culture and the expansion of epithelial cell population at passage 3. (c, d) Mesenchymal-type cells in the primary culture at 10–15 days and after culturing to elongated spindle-shaped or flat “stromal” cell populations at passage 3.

**Figure 2:**
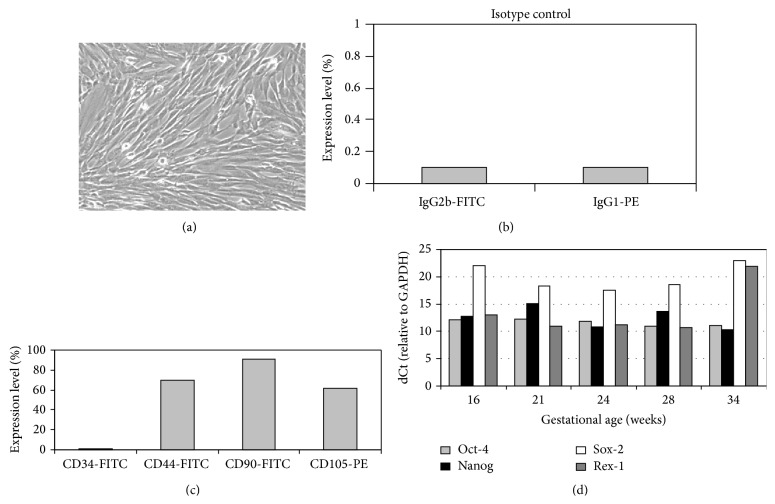
Phenotypical characteristics of AF-MSCs from late trimesters. (a) Representative image of spindle-shaped MSCs at passage 4. (b) Expression of cell surface markers from representative FACS histograms of isotype negative controls and (c) AF-MSCs gated for CD34, CD44, CD90, and CD105. (d) The relative expression of pluripotency markers Oct-4, Nanog, Sox-2, and Rex-1 in AF-MSCs populations obtained from amniocentesis samples of second- and third-trimester at passages 4–6 by RT-qPCR. The expression of mRNA was normalized to GAPDH.

As reported previously [9, 24], the amniotic fluid cells represent a heterogeneous population and only 1% of them demonstrate stem cell characteristics. In this study, a two-step cultivation protocol [22] was used for producing AF cell population with homogeneous fibroblast-like morphology. Adherent cells derived from fresh AF samples of late second- and third-trimester in primary culture resulted in a mixed population with spindle- and round-shaped morphology forming colonies at about 15–20 days of culture (Figure 1(a)). The colonies of small and spherical cells with a centrally located nucleus resemble epithelial cells that form islands in culture of 2-3 passages (Figure 1(b)). The primary culture contained a slowly growing cell population, which displayed large and flat “stromal” cells with irregular cytoplasmic extensions and very small nucleus at the edge of cytoplasm (Figure 1(d)). Spindle-shaped cells of fibroblast-like morphology with a high proliferation potential were predominantly present in culture after the second and third passage as well (Figure 1(c)). The derivation of mesenchymal stem cell population using two-step protocol was successful, and after 4–8 passages in culture, the population became morphologically homogeneous with fibroblastic morphology (Figures 1(c) and 2(a)). These AF-MSCs grew to 80–90% confluence of the subsequent passage culture in 2–4 days and were tested on their cellular phenotypic characteristics and differentiation potential at passages 4–6.

The characteristics of cell surface markers of AF-MSCs were determined by flow cytometry (Figures 2(b) and 2(c)). The results showed that MSCs from different AF samples (*n* = 12) expressed the adhesion molecule CD44 (from 53.9 to 69.9%), high levels of CD90 (Thy-1) (from 69.9 to 91.2%), and mesenchymal-related antigen CD105 (endoglin) (from 49.3 to 61.6%) and were negative for hematopoietic stem cell marker CD34. Of note, the expression levels of “stemness” markers (CD44 and CD105) were higher in MSCs populations with more characteristic mesenchymal stem cell morphology and phenotype and those populations expressed higher levels of CD90, which might be related to the growth rate of AF-MSCs [8].

MSCs from AF of different gestation age (16–28 weeks) were evaluated for the expression of stem cell pluripotency markers (Figure 2(d)). Results of Q-RT-PCR analysis demonstrated that MSCs at 4–6 passages consistently expressed* Oct-4, Nanog, Sox-2,* and* Rex-1*, which are associated with the maintenance of the undifferentiated state and the pluripotency. AF-MSCs from second- and third-trimester (16–28 weeks) expressed comparable levels of* Oct-4* and* Nanog*, while some difference in the expression levels of* Sox-2* and* Rex-1* was related to the gestational age. MSCs from late trimester of gestation (34 weeks) exhibited lower expression of both* Sox-2* and* Rex-1*. MSCs induced to multipotent differentiation did not express these genes (data not shown).

### 3.2. Differentiation Potential of AF-MSCs

**Figure 3:**
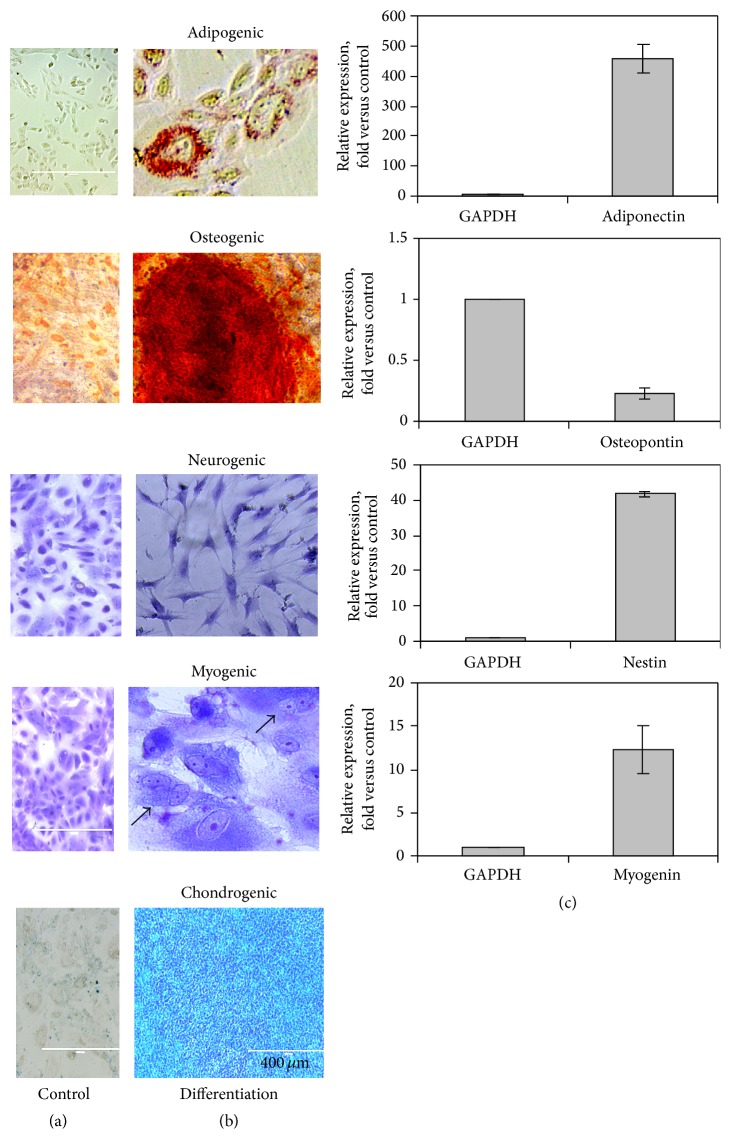
Differentiation potential of AF-MSCs. AF-MSCs were obtained from amniocentesis samples of second- and third-trimester at passages 5–8, cultured without differentiation supplements (a), or maintained in differentiation media. (b) Representative images of AF-MSCs after adipogenic treatment showing the accumulation of lipid vacuoles by Oil Red O staining; osteogenic treatment by Alizarin Red staining for calcium mineralization; neurogenic or myogenic treatment showing the presence of neuron-like cells or multinucleated cells by staining with crystal violet, respectively, and chondrogenic treatment showing glycosaminoglycan production in chondrogenic pellets by Alcian Blue staining. (c) Relative expression of* adiponectin, myogenin, nestin, and osteopontin* by RT-qPCR is presented as* n*-fold increase over untreated control. Data are presented as the mean ± S.N. (*p* < 0.05) for three independent experiments.

Furthermore, AF-MSCs obtained from amniocentesis samples of different gestational time were analysed for their capacity to differentiate toward adipogenic, osteogenic, chondrogenic, myogenic, and neurogenic lineages. All clones of AF-MSCs from amniocentesis samples of second- (16–19 week) and third-trimester (34 week) that grew in culture beyond 4 or 8 passages were able to differentiate in all lineages tested (Figure 3). Cells cultured under adipogenic condition for 12 days accumulated lipid vacuoles and exhibited intense staining with Oil Red O (Figure 3(b)). RT-qPCR analysis showed very high expression level (456-fold increase of control) of adipocytes marker* adiponectin* (Figure 3(c)). Similarly, after culturing with osteogenic medium for 12 days, most of cells exhibited extracellular matrix mineralization detected by Alizarin Red S staining. In this cell culture, the analysis of osteogenic transcript levels determined by RT-qPCR showed unexpected low expression of* osteopontin* relative to untreated control, expressing this gene too. At 6–12 days after neural induction, morphologically neural-like cells were observed by light microscope after staining with 0.1% crystal violet. In differentiated cells, the expression level of* Nestin* detected by RT-qPCR analysis was upregulated (28-fold increase) relative to untreated control, where* Nestin* is expressed as well. Myogenic differentiation was obvious by the presence of multinucleated cells as determined by phase contrast microscope after staining with 0.1% crystal violet (Figure 3(b)) and the expression of* myogenin* (12,3-fold increase) detected by RT-qPCR (Figure 3(c)). For the induction of chondrogenic differentiation, MSCs were cultured in high-density pellet mass culture. Chondrogenic differentiation was determined after 20 days by the appearance of chondrogenic pellet and the glycosaminoglycan production detected by Alcian Blue staining. Cultured AF-MSCs (control) did not show any of the above differentiation morphology (Figure 3(a)).

### 3.3. Proteome Differences Associated with Myogenic, Adipogenic, Osteogenic, and Neurogenic Differentiation of Late Second-Trimester AF-MSCs

**Figure 4:**
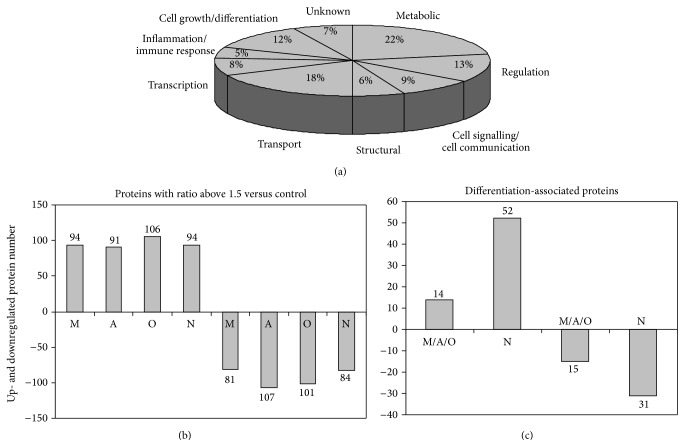
Differentially expressed proteins in AF-MSCs of second-trimester undergoing myogenic (M), adipogenic (A), osteogenic (O), and neurogenic (N) differentiation. (a) Venn diagram depicting functional classification of differentiating cell proteins expressed 1.5-fold above control. (b) Number of up- and downregulated proteins and (c) differentiation-associated proteins with ratio 1.5 versus control in each differentiating population.

**Figure 5:**
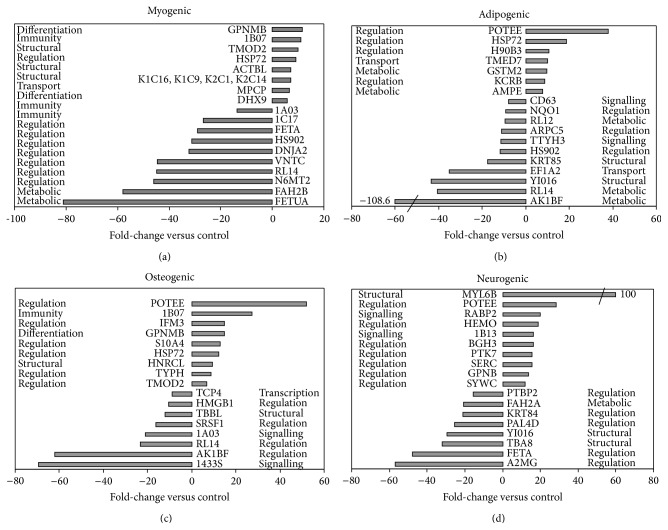
Highly up- and downregulated proteins in second-trimester AF-MSCs undergoing (a) myogenic, (b) adipogenic, (c) osteogenic, and (d) neurogenic differentiation. Each bar corresponds to the expression fold differences higher than six or lower than eight in each differentiating population as compared with undifferentiated AF-MSCs.

For a comparison of the proteome profile, we presented a sample of normal cultured AF-MSCs from second-trimester (16 weeks, passage 5), cultured under appropriate conditions to induce myogenic, adipogenic, osteogenic, and neurogenic differentiation. The SYNAPT G2 High Definition Mass Spectrometry based analysis demonstrated 1423 proteins expressed in AF-MSCs. The relative protein expression ratios were calculated for each differentiation, and proteins with the expression ratio above 1.5 compared with undifferentiated control were selected and presented in Supplementary Table 1 (see Supplementary Table 1 in Supplementary Material available online at http://dx.doi.org/10.1155/2015/319238). The abbreviated and full names of the proteins, the accession numbers, the theoretical molecular weight and pI values, Max score and the number of reported peptides, and the percentage coverage of the identified proteins are listed in the table.  Figure 4(a) represents the functional classification of 250 selected proteins from AF-MSCs differentiated toward myogenic, adipogenic, osteogenic, and neurogenic lineages with the expression ratio above 1.5 versus control. The groups of selected proteins were related to cell growth and differentiation (12%), regulation (13%), cell signalling/communication (9%), and transcription/translation (8%). Other groups represent metabolic (22%), transport (18%), structural (6%), and immune response (5%) proteins, including 7% of unknown proteins. Among 250 of high abundance proteins selected (Figure 4(b)), 91, 89, 96, and 87 were upregulated and 89, 105, 106, and 81 downregulated in AF-MSCs undergoing myogenic, adipogenic, osteogenic, and neurogenic differentiation, respectively. A number of proteins were absent in undifferentiated AF-MSCs, including latexin (LXN, involved in negative regulation of peptidase activity), growth differentiation factor 6 (GDF6, involved in multicellular organism development), Ig mu heavy chain disease protein (MUCB, an extracellular vesicular exosome), alpha crystallin B chain (CRYAB, involved in metabolic processes), glycogen synthase kinase 3 beta (GSK3*β*, involved in glycogen metabolism and signalling), and ATP dependent RNA helicase DDX 19A (DD19A, involved in many biological, metabolic, and transport processes) (Supplementary Table 1). Next we identified significant changes in the expression levels of up- and downregulated proteins in differentiated cells compared with undifferentiated control (Figure 5).

During myogenic differentiation (Figure 5(a)), the highest expression ratios were found for such proteins: transmembrane glycoprotein NMB (GPNMB, ratio 11.7, involved in negative regulation of cell proliferation and cell adhesion), HLA class I histocompatibility antigen B7 alpha chain (1B07, ratio 11.2, expressed in nearly all cells), tropomodulin 2 (TMOD2, ratio 10.0, involved in regulation of actin filaments in muscle and nonmuscle cells), heat shock related 70 kDa protein 2 (HSP72, ratio 9.2, involved in signal transduction, mitotic cell cycle regulation), the family of keratin type I/II cytoskeletal (K1C9, K1C14, K1C16, K2C14, ratios 7.9–5.8, involved in cytoskeletal organization and cell differentiation), *β*-actin-like protein 2 (ACTBL, ratio 7.3, a muscle tissues cytoskeleton component), phosphate carrier protein mitochondrial (MPCP, ratio 6.6, catalyses the transport of phosphate into the mitochondrial matrix), and ATP dependent helicase (DHX9, ratio 5.7, involved in cell growth, division, and differentiation). The expression of nine proteins, which regulate different cellular processes, was significantly downregulated (ratio 13.8–58) as well as additional 20 proteins with ratio above 5 (Supplementary  Table  1).

During adipogenic differentiation (Figure 5(b)), seven regulatory proteins, that is, POTE ankyrin domain family member E (POTEE, expressed in ES cells with a specific function during lineage-specific differentiation), heat shock related 70 kDa protein 2 (HSP72), putative heat shock protein HSP90 beta 3 (H90B3, molecular chaperone to promote the maturation, structural maintenance, and proper regulation of specific target proteins), creatine kinase B type (KCRB, expressed by various tissues and cell types) with ratios 37.9, 18.8, 10.6, and 8.6, respectively, and proteins having a metabolic role, such as glutathione S transferase Mu 2 (GSTM2) and glutamyl aminopeptidase (AMPE) with ratios 9.8 and 7.9, respectively, or transport function, such as transmembrane emp24 domain containing protein (TMED7, ratio 10.0), were highly expressed. Ten proteins were highly downregulated (with ratios 8.0–40.6), while the expression of aldo-keto reductase family 1 member B15 (AF1BF, involved in oxidation-reduction process) decreased 108.6 times compared with control.

For osteogenic differentiation (Figure 5(c)), the expression of ten proteins was significantly higher than that of control: POTEE, ratio 51.9; HLA class I histocompatibility antigen B7 alpha chain (1B07, ratio 27.4); interferon induced transmembrane protein 3 (IFM3, ratio 14.9); transmembrane glycoprotein NMB (GPNMB, ratio 14.8, involved in osteoblast differentiation, bone mineralization); protein S100 4A (S10A4, ratio 12.9, induces the expression and secretion of osteopontin); HSP72 with ratio 12.4; heterogeneous nuclear ribonucleoprotein C-like 1 (HNRCL, ratio 9.3), thymidine phosphorylase (TYPH, ratio 8.6); tropomodulin 2 (TMOD2, ratio 6.8, involved in actin filament organization). Two proteins were highly downregulated, that is, aldo-keto reductase family 1 member B15 (AF1BF, ratio 62.0, involved in oxidation-reduction processes) and 14 3 3 protein sigma (14 3 3S, ratio 64.3, which have the ability to bind a multitude of functionally diverse signalling proteins). The expression levels of six proteins decreased by 8.8–23.4-fold (Figure 5(c)) and additional six proteins by 5.9–7.9-fold (Supplementary Table 1).

During neurogenic differentiation, eight proteins with different functional roles were highly downregulated (by 15.6–56.8-fold) (Figure 5(d)). Among them, significant decreases in the expression were determined for alpha 2 macroglobulin (A2MG, ratio 56.8, involved in regulation of many growth factors and cytokines), alpha fetoprotein (FETA, ratio 47.9, involved in the foetus protection from maternal estradiol), and tubulin alpha 8 chain (TBA8), ratio 31.9, involved in regulation of the assembly and dynamics of axonemal microtubules.

Specific proteins were identified in AF-MSCs differentiated to neurogenic lineage, including selenium binding protein 1 (SBP1), which is exclusively located at the growing tips of neurites; secreted frizzled related protein 1 (SFRP1), involved in downregulation of Wnt signalling; metalloproteinase inhibitor 3 (TIMP3), which complexes with metalloproteinase (such as collagenases) and irreversibly inactivates them; integrator complex subunit 4-like protein 2 (IN4L2) of unknown function (Supplementary Table 1). Proteins in neuronal cells are presented in Figure 5(d), which are involved in signalling and regulation processes, and were highly upregulated, namely, myosin light chain 6B (MYL6B, ratio 100, the ATPase cellular motor protein); POTEE with ratio 28.4, cellular retinoic acid binding protein 2 (RABP2, ratio 20.1, involved in retinoid signalling pathway); hemopexin (HEMO, ratio 19.0, which protects from oxidative damage); HLA class I histocompatibility antigen B 13 alpha chain (1B13, ratio 16.3); transforming growth factor beta inducible protein ig h3 (BGH3, ratio 16.3, involved in cell differentiation); inactive tyrosine protein kinase 7 (PTK7, ratio 15.6, involved in a positive regulation of neuron projection); phosphoserine aminotransferase (SERC, ratio 15.5, involved in serine biosynthesis); phosphoserine aminotransferase (GPNB, ratio 13.7, expressed in dendritic cells); tryptophan tRNA ligase cytoplasmic (SYWC, ratio 11.9, which have multifunctional regulatory role). Proteins associated with neurogenic differentiation, which displayed a significant increase in their expression with ratios 1.5–8 higher than control, were detected also (Table 1(b)), including integrin alpha 8 (ITA8, ratio 7.2, involved in nervous system development); seprase (ratio 6.6, which participate in epithelial-mesenchymal interaction in development control); tenascin (ratio 4.69, involved in nervous system development); HLA class I histocompatibility antigen, B7 alpha chain (ratio 1.88, involved in regulation of dendritic cell differentiation); protein arginine N-methyltransferase 1 (ratio 1.76, involved in neuron projection development); polypyrimidine tract binding protein 2 (ratio 15.6, found in neuronal-derived cells). Also other upregulated proteins associated with neurogenic differentiation were identified: integrin 19 alpha 11 (ratio 2.39), prosaponin (ratio 2.34), cadherin 13 (ratio 2.5), protein S100 A6 (ratio 2.5), calponin 3 (ratio 2.1), neural cell adhesion molecule L1 (ratio 1.53), and nestin (ratio 1.5).

The analysis by UniprotKB/SwissProt human database and the DO classification of the proteome was used to determine the significant changes in proteins associated with cell differentiation (Supplementary Table 1). Based on these results, the number of proteins with differential expression by ratio above 1.5 (1st group for myogenic, adipogenic, and osteogenic differentiation) or above 2 (2nd group for neurogenic differentiation) compared with control is shown in Figure 4(c). In the 1st group, 14 proteins were upregulated and 15 proteins were downregulated, while neurogenic differentiation exhibited a higher number of such changes in identified proteins expression (52 and 31, resp.). Among differentiation-related proteins (Table 1(a)), glycogen synthase kinase 3 beta and cyclin dependent kinase 1 were present only in differentiated cells of the 1st group, while integrin alpha 8 was found only in 2nd group (Table 1(b)). In both groups, the expression of proteins, such as the growth differentiation factor 6, 3-hydroxybutyrate dehydrogenase type 2, tropomyosin alpha chain, annexin A 4, protein arginine N methyltransferase 1, guanine nucleotide binding protein subunit alpha 11, and mitogen activated protein kinase 1, was upregulated. High expression of GPNMB was characteristic for myogenic, osteogenic, and neurogenic differentiation, while DD19A, GNA11 are for myogenic, adipogenic, and osteogenic differentiation and K1C14 is only for myogenic differentiation. Table 1(a) also represents downregulated proteins associated with differentiation toward four lineages; some of them, including AINX and BGH3, were upregulated during neurogenic differentiation. This type of differentiation showed the largest relative expression change for proteins with ratios above 2–7.45. The expression of ERAP1, ITA8, WNT5A, CO1A1, TENA, PML, and RRAS was 7.45–4.64-fold higher in cells differentiated to neurogenic lineage compared with undifferentiated control (Table 1(b)).

We also identified proteins with increased expression levels, which are known as markers of lineage specificity in differentiated cells. A summary of the expression of markers for myogenic, adipogenic, osteogenic, and neurogenic differentiation is shown in Table 2. Myogenic precursor markers (integrin alpha 5, caveolin 1), smooth muscle markers (desmin, calponin 1, transgelin 3, caldesmon, and myosin light chain kinase smooth muscle), and muscle cell structural proteins (tropomyosin alpha 4 chain, myosin 10, and myosin regulatory light chain 12A) were found upregulated with a mean fold-change of 1.2–3.3 versus undifferentiated control. The expression of adipocyte markers, such as cellular retinoic acid binding protein 2, peroxisomal multifunctional enzyme type 21, and adipocyte plasma membrane associated protein, was 1.3–2.4-fold higher in differentiated cells compared with control. Tropomyosin alpha 4 chain, collagen *α* 3–6 chain, casein kinase II subunit alpha, and matrix metalloproteinase 14, which are associated with osteogenic differentiation, displayed also increased ratios (1.2–2.5 versus control) in differentiated cells. For neurogenic markers, calponin 3, cadherin 13, tubulin beta 3 chain, and nestin were upregulated with ratios 1.2–2.5 versus control.

The examples of several proteins (Supplemental Table 1) expressed in high amounts in cultured AF-MSCs, which were downregulated (by about 1.2–2-fold) in cells undergoing differentiation to distinct lineages, included vimentin (a developmental marker of MSCs), galectin 1 (stem cell regulatory molecule), proliferation-associated protein 2G4 (involved in MSCs proliferation and maintenance), gelsolin (calcium-regulated, actin-modulating protein), cofilin 1 (intracellular actin-modulating protein), transgelin (actin-binding protein with contractile properties), and protein disulfide isomerase (involved in protein folding and redox signalling). Proteins upregulated (by about 1.2–2.4-fold) in cultured AF-MSCs compared to differentiated cells included prohibitin (involved in cell development), chloride intracellular channel protein 4 (involved in cellular processes regulation), protein enabled homolog (involved in cell structure reorganization), or Rho GDP dissociation inhibitor 1 (involved in cell motility and signalling).

## 4. Discussion

In the presented study, we explored AF-SCs mesenchymal characteristics and multilineage potential from second- and third-trimester of gestation. We demonstrated that AF-MSCs isolated by two-stage culture method are fibroblastic F-type cells of mesenchymal origin with phenotypic characteristics of stem cells. Those cells that grew beyond 5–8 passages expressed mesenchymal cell markers (CD44, CD90, and CD105) and did not express a marker of hematopoietic cell phenotype (CD34). Here, we found some variations in the expression of CD44 and CD105 between AF samples associated probably with the purity of the population tested because of the existence of a small part of morphologically and phenotypically distinct cells among the abundant mesenchymal cell type cells. As shown previously, AF cell population, enriched with CD44+/CD105+ cells and the expression of “stemness” markers, exhibited higher proliferation rates than CD44−/CD105− population [8]. In our study, high expression levels of CD90 were found in all AF samples, which were strongly positive for the embryonic stem cell characteristic markers (Oct-4, Sox-2, Rex-1, and Nanog) that are associated with the maintenance of the undifferentiated state and the pluripotency as was demonstrated previously [13, 16, 24, 25]. Importantly, these cells were able to differentiate toward adipocytes, osteoblasts, chondroblasts, myocytes, and neural-like cells. Additionally, we observed that third-trimester AF-MSCs had lower differentiation potential to myocytes and stronger to neuron-like cells. Our data confirm a recent report [26], which describes that among isolated AF-SCs populations from third-trimester several cultures expressed neuronal and glial markers, including* nestin*, indicating their potential attitude toward a neural fate but poor capacity to differentiate into adipogenic and osteogenic lineages. Moreover, nestin not only has been found in our AF-SCs samples, but also was found abundant in embryonic stem-derived progenitor cells, representing a characteristic marker of neuroepithelial stem cells [27]. Other studies [15, 26, 28] also argue for the existence of neural progenitor cells in second-trimester AF from normal pregnancies. Furthermore, our data indicated the possibility of AF-MSCs to differentiate to myocytes. The previous study [19] demonstrated that AF stem cells have a potency to differentiate to a cardiomyogenic phenotype.

Here we found that MSCs from second- and even third-trimester expressed comparable levels of factors of pluripotency and self-renewal, Nanog and Oct-4. This is consistent with the reports that cells isolated from early gestational weeks [1, 3, 8] as well as from late mid-trimester [21] express the markers of pluripotency, Oct-4 and Nanog, although decreased Oct-4 expression has been found in AF cell cultures between 15 and 22 weeks of gestation at 8–10 passages [29].

As was shown, isolated AF-MSCs clones could be expanded in culture to 15–20 or 10 passages at gestational age of 15-16 weeks or 18–20 weeks, respectively [29]. An inverse correlation exists between culture duration and gestational age. Slow growth of late gestational age AF cells may be caused by aneuploid karyotypes, translocations, and inversions [30]. Our data indicated that MSCs from amniocentesis samples of every trimester studied and expanded in culture retained the proliferation and differentiation potential during 5–8 passages. It has been demonstrated [11, 25] that c-kit positive cells, which constitute only 1–5% of the total AF cells, are broadly multipotent, although the amount of those cells increased between 16 and 22 weeks of gestation, but later disappeared [3, 12]. Recently, it is suggested that first- and second-trimester AF-SCs are related, but distinct populations. First-trimester AFS cells are smaller and have higher growth rate than those of second-trimester [31]. Microarray-based transcriptome analysis of C-kit positive population in AF-SCs showed a common gene expression profile (88.8%) but the unique cell-specific gene expression. First-trimester AF-SCs are more undifferentiated phenotype of pluripotent cells and express a larger number of organ-specific genes, while second-trimester AF-MSCs exhibit higher expression levels of genes involved in specifications of cells and tissues, but some genes involved in the pluripotency are switched off [31].

For the first time, our study provides evidence of differentially expressed protein profiles in AF-MSCs undergoing myogenic, adipogenic, osteogenic, and neurogenic differentiation. The SYNAPT G2 High Definition Mass Spectrometry was applied to define changes in the proteome more accurately. To date, a limited number of proteomic studies was performed on human amniotic fluid-derived MSCs. The amniotic fluid cell extract, containing E-type, AF-type, and F-type cells, was analysed and 2-dimensional database revealed 432 different gene products (2400 spots) [32]. The variation in the expression of 23 proteins that occur in early and late passages of cultured CD117^+^ AF-MSCs was demonstrated by proteomic analysis [33]. Using the 2-dimensional gel electrophoresis and MALDI-TOF/MS approach, the comparative analysis of proteomic maps of cultured AF-MSCs from amniocentesis between the 15th and 18th weeks of gestation and bone marrow-derived MSCs was performed by Roubelakis et al. [14], where 261 different proteins were identified with the similar functional proteomic pattern derived from both sources. Among them, 137 proteins were present only in AF-MSCs; 78 proteins were unique and related to proliferation and primitive phenotype. Later, the authors [8] established a comparative proteomic map of two morphologically different adherent cell types (spindle-shaped and round-shaped) from second-trimester, identifying 25 differentially expressed proteins in the two populations. The results of these studies helped us with the identification of changes in lineage-specific protein expression, presented in Table 2. Based on GO classification, our study revealed differentiation-related proteins, which were up- or downregulated differentially in cells undergoing differentiation to distinct lineages (Tables 1(a) and 1(b)). Moreover, we found about 20 different proteins strongly down- and overexpressed (above 6, 10 and more fold versus control) in the four differentiated populations (Figure 5), which participate in many cellular processes, including proteins related to specific differentiation, such as TMOD2, K1C9, K1C14, K1C16, and K2C14 for myogenic differentiation; GPNMB, S10A4 for osteogenic differentiation; PTK7, GPNB, ITA8, and WNT5A for neurogenic differentiation. Additionally, we identified the changes in the expression of proteins related to undifferentiated AF-MSC state and known as specific differentiation markers [24]. In general, proteomic analysis demonstrated the similar proteome of undifferentiated and differentiated AF-MSCs, and only six proteins were not present in cultured AF-MSCs, while about 215 from 250 of high abundance proteins selected from more than 1400 were found up- or downregulated during the differentiation toward distinct lineages.

## 5. Conclusions

The results presented here demonstrate that healthy women amniotic fluid MSCs from late second- and third-trimester displayed similar mesenchymal stem cell characteristics related to morphology, proliferation capacity, expression of specific cell surface and pluripotency markers, and multilineage differentiation potential. Using SYNAPT G2 High Definition Mass Spectrometry technique approach, we identified proteomic profiles of cultured AF-MSCs from late trimester of gestation and differentiated toward four distinct lineages. The detailed comparative proteomic analysis of 250 proteins selected from more than 1400 proteins led to clarify the differences in the expression of specific proteins in AF-MSCs undergoing differentiation that may facilitate the studies for the elucidation of the molecular profiles and identification of key markers expressed in those cells.

## Supplementary Material

Proteome differences associated with myogenic, adipogenic, osteogenic, and neurogenic differentiation of late second-trimester AF-MSCs are presented in Supplementary Table 1 (see Supplementary Table 1 in Supplementary Material available online at http://dx.doi.org/10.1155/2015/319238). For a comparison of the proteome profile, we presented a sample of normal cultured AF-MSCs from second-trimester (16 weeks, passage 5), cultured under appropriate conditions to induce myogenic, adipogenic, osteogenic, and neurogenic differentiation. The SYNAPT G2 High Definition Mass Spectrometry based analysis demonstrated 1423 proteins expressed in AF-MSCs. The relative protein expression ratios were calculated for each differentiation, and proteins with the expression ratio above 1.5 compared with undifferentiated control were selected and presented in Supplementary Table 1.

## Figures and Tables

**(a):** 

GO term name	Entry	Protein full name	Fold-changes versus control			
			M/C	A/C	O/C	N/C
Cell differentiation	CDK1_HUMAN	Cyclin dependent kinase 1	***+/−***	***+/−***	***+/−***	***+/−***
Neuron differentiation	GDF6_HUMAN	Growth differentiation factor 6	***+/−***	***+/−***	***+/−***	***+/−***
Fat cell, myotubule differentiation	GSK3B_HUMAN	Glycogen synthase kinase 3 beta	***+/−***	***+/−***	***+/−***	***−/−***
Osteoblast differentiation	GPNMB_HUMAN	Transmembrane glycoprotein NMB	11.71	**0.03**	14.80	13.73
Epithelial cell differentiation	K1C14_HUMAN	Keratin type I cytoskeletal 14	6.75	**0.65**	**0.40**	**0.82**
Osteoblast differentiation	DD19A_HUMAN	ATP dependent RNA helicase	5.68	6.05	3.2	0.61
Osteoblast differentiation	GNAS1_HUMAN	Guanine nucleotide binding protein G s subunit alpha isoforms	3.14	2.44	1.73	0.14
Epithelial cell differentiation	BDH2_HUMAN	3-Hydroxybutyrate dehydrogenase type 2	2.01	1.93	2.0	2.81
Osteoblast differentiation	TPM4_HUMAN	Tropomyosin beta 4 chain	1.86	1.85	2.07	1.08
Epithelial cell differentiation	ANXA4_HUMAN	Annexin A4	1.74	1.99	1.82	2.52
Megakaryocyte differentiation	ANM1_HUMAN	Protein arginine N methyltransferase 1	1.72	2.26	1.76	1.14
Cell differentiation	L1CAM_HUMAN	Neural cell adhesion molecule L1	1.59	**0.4**	1.54	0.97
Melanocyte differentiation	GNA11_HUMAN	Guanine nucleotide binding protein subunit 11	1.58	1.65	1.88	1.99
Cell differentiation	MK01_HUMAN	Mitogen activated protein kinase 1	1.56	2.01	1.86	1.07
Osteoblast differentiation	PHB_HUMAN	Prohibitin	0.55	0.48	0.49	**1.12**
Stem cell differentiation	A2MG_HUMAN	Alpha 2 macroglobulin	0.44	0.26	0.37	0.02
Cell differentiation	AINX_HUMAN	Alpha internexin	0.39	0.26	0.56	**1.43**
Melanocyte differentiation	GBLP_HUMAN	Guanine nucleotide binding protein subunit beta-2-like 1	0.36	0.47	0.3	0.73
Osteoblast differentiation	RS15_HUMAN	40S ribosomal protein S15	0.36	0.56	0.28	0.98
Neuron, muscle differentiation	CSN2_HUMAN	COP9 signalosome complex subunit 2	0.34	0.55	0.33	0.81
Cell differentiation	RS3A_HUMAN	40S ribosomal protein S3a	0.33	0.46	0.21	0.76
Erythrocyte differentiation	RS14_HUMAN	40S ribosomal protein S14	0.33	0.47	0.18	0.67
Cell differentiation	BGH3_HUMAN	Transforming growth factor beta-induced protein ig-h3	0.33	0.26	0.16	**16.27**
Cell differentiation	MP2K1_HUMAN	Dual specificity mitogen activated protein kinase 1	0.3	0.5	0.52	0.36
Erythrocyte differentiation.	RS19_HUMAN	40S ribosomal protein S19	0.29	0.42	0.23	0.83
Cell differentiation	CYR61_HUMAN	Protein CYR61	0.22	0.43	0.17	0.39
Keratinocyte differentiation	KRT84_HUMAN	Keratin type II cuticular Hb4	0.04	0.42	0.39	0.05
Endodermal differentiation	VTNC_HUMAN	Vitronectin	0.02	0.49	0.16	0.59

**(b):** 

GO term name	Entry	Protein full name	Fold-changes vs. control
			N/C
Fat cell differentiation	ERAP1_HUMAN	Endoplasmic reticulum aminopeptidase 1	7.45
Cell differentiation	ITA8_HUMAN	Integrin alpha 8	7.20
Neuron, fat cell differentiation	WNT5A_HUMAN	Protein Wnt-5a	6.49
Endodermal cell differentiation	ITAV_HUMAN	Integrin alpha 5	4.75
Osteoblast differentiation	CO1A1_HUMAN	Collagen alpha 1 (I) chain	6.12
Osteoblast differentiation	TENA_HUMAN	Tenascin	4.69
Myeloid cell differentiation	PML_HUMAN	Protein PML	4.64
Fat cell differentiation	SODM_HUMAN	Superoxide dismutase [Mn] mitochondrial	3.94
Leukocyte differentiation	RRAS_HUMAN	Ras-related protein R-Ras	4.31
Cell differentiation	TYPH_HUMAN	Thymidine phosphorylase	3.22
Osteoblast differentiation	MRC2_HUMAN	C-type mannose receptor 2	3.16
Epithelial, myeloid cell differentiation	IF16_HUMAN	Gamma interferon-inducible protein 16	3.58
Epithelial cell differentiation	TPP1_HUMAN	Tripeptidyl-peptidase 1	3.54
Dendritic cell differentiation	HLAG_HUMAN	HLA class histocompatibility antigen, alpha chain G	3.14
Epithelial cell differentiation	AK1C1_HUMAN	Aldo-keto reductase family 1 member C1	3.11
Neuron, astrocyte, and glial cell differentiation	STAT3_HUMAN	Signal transducer and activator of transcription 3	2.91
T cell differentiation	B2MG_HUMAN	Beta-2 microglobulin	2.65
Erythrocyte differentiation	ADDA_HUMAN	Alpha adducin	2.59
Epithelial cell differentiation	GNA11_HUMAN	Guanine nucleotide binding protein subunit alpha 11	2.58
Epithelial cell differentiation	CBR1_HUMAN	Carbonyl reductase [NADPH] 1	2.58
Cell differentiation	FHL1_HUMAN	Four and a half LIM domains' protein 1	2.58
Erythrocyte differentiation	CSRP2_HUMAN	Cysteine and glycine-rich protein 2	2.58
Osteoblast differentiation	ITA11_HUMAN	Integrin alpha 11	2.39
Epithelial cell differentiation	SAP_HUMAN	Prosaposin	2.34
Chondrocyte differentiation	GSLG1_HUMAN	Golgi apparatus protein 1	2.22
Nervous system differentiation	CAD13_HUMAN	Cadherin 13	2.5
Cell differentiation	S10A6_HUMAN	Protein S100 A6	2.5
Endodermal cell differentiation	CO8A1_HUMAN	Collagen alpha 1–VIII chain	2.15
Epithelial cell differentiation	CNN3_HUMAN	Calponin 3	2.10
Cell differentiation	FLNB_HUMAN	Filamin-B	2.10
Endodermal cell differentiation	SFRP1_HUMAN	Secreted frizzled related protein	2.06

**Table 2:** Lineage-specific proteins upregulated in AF-MSCs undergoing myogenic (M), adipogenic (A), osteogenic (O), and neurogenic (N) differentiation. A mean ratio above 1.3 is defined as upregulated expression in differentiated cells.

Protein full name	Fold-changes versus control	Remarks
Ratio M/C		
Desmin	3.3	Smooth muscle marker
Calponin 1	2.8	Smooth muscle marker
Transgelin 3	2.0	Smooth muscle marker
Tropomyosin alpha 4 chain	1.9	Muscle cell contraction regulation
Integrin alpha 5	1.8	Myogenic precursor marker
Myosin light chain kinase smooth muscle	1.7	Smooth muscle marker
Myosin 10	1.7	Actomyosin structure organization
Myosin regulatory light chain 12A	1.6	Muscle cell contraction regulation
Caveolin 1	1.5	Myogenic precursor marker

Ratio A/C		
Cellular retinoic acid binding protein 2	2.4	Expressed in preadipocytes
Peroxisomal multifunctional enzyme type 21	1.7	Involved in fatty acid, lipid metabolism
Adipocyte plasma membrane associated protein	1.3	Expressed in adipocytes

Ratio O/C		
Tropomyosin alpha 4 chain	2.1	Osteoblast differentiation
Collagen alpha 3–6 chain	2.5	Matrix component organization
Casein kinase II subunit alpha	1.4	Cellular processes regulation

Ratio N/C		
Cadherin 13	2.5	Adhesion in nervous system
Calponin 3	2.1	Actomyosin structure organization
Nestin	1.5	Neuronal progenitor cell marker
Tubulin beta 3 chain	1.3	Required for axon growth/guidance

## Abbreviations

AF-MSCs: Amniotic fluid-derived mesenchymal stem cells
HDMS: High Definition Mass Spectrometry
GAPDH: Glyceraldehyde-3-phosphate dehydrogenase
Oct-4: Octamer-binding transcription factor 4
RT-qPCR: Quantitative real-time polymerase chain reaction
Sox-2: Sex determining region Y-box 2.
