# Supplementary material for: Human Amniotic Fluid Mesenchymal Stem Cells from Second- and Third-Trimester Amniocentesis: Differentiation Potential, Molecular Signature, and Proteome Analysis
Source: Stem Cells Int. 2015 Aug 17;2015:319238. doi: 10.1155/2015/319238 (PMC4553339; doi:10.1155/2015/319238)
Supplement: Supplementary file 1 — Proteome differences associated with myogenic, adipogenic, osteogenic, and neurogenic differentiation of late second-trimester AF-MSCs are presented in Supplementary Table 1 (see Supplementary Table 1 in Supplementary Material available online at http://dx.doi.org/10.1155/2015/319238). For a comparison of the proteome profile, we presented a sample of normal cultured AF-MSCs from second-trimester (16 weeks, passage 5), cultured under appropriate conditions to induce myogenic, adipogenic, osteogenic, and neurogenic differentiation. The SYNAPT G2 High Definition Mass Spectrometry based analysis demonstrated 1423 proteins expressed in AF-MSCs. The relative protein expression ratios were calculated for each differentiation, and proteins with the expression ratio above 1.5 compared with undifferentiated control were selected and presented in Supplementary Table 1. [file 319238.f1.pdf]

SUPPLEMENT

TABLE 1: Proteins expressed in AF-MSCs and identified by SYNAPT G2 HD Mass Spectrometer. Proteins with the expression ratio above 1.5 in comparison with undifferentiated control were selected.

A mean folde change in the expression of proteins is presented as the median of Myo/C, Adipo/C, Osteo/C, Neuro/C ratios calculated between myogenically (Myo), adipogenically (Adipo), osteogenically (Osteo) and neurogenically (Neuro) differentiated and undifferentiated (C) cells. A positive (+) protein expression is defined vs. negative (-) expression in undifferentiated cells. Statistically significant differences of protein expression levels were determined using Student's t-test and presented in the table as P (Myo/C, Adipo/C, Osteo/C, Neuro/C) where ≥ 95 (up-regulation) arba ≤ -95 (down-regulation).

Nr.	Supplemental Table Description	Entry	Accession number	pI	MW	Max score	Reported peptides	Sequence coverage, %	Ratio Myo/C	Ratio Adipo/C	Ratio Osteo/C	Ratio Neuro/C	P Myo/C	P Adipo/C	P Osteo/C	P Neuro/C
1	Latexin OS Homo sapiens GN LXN PE 1 SV 2	LXN_HUMAN	Q9BS40	5.42	25864	10966.13	2	12.16	**+**	**+**	**+**	**+**	88.36	100.00	99.98	100.00
2	Growth differentiation factor 6 OS Homo sapiens GN GDF6 PE 1 SV 1	GDF6_HUMAN	Q6KF10	9.01	51175	806.42	2	12.31	**+**	**+**	**+**	**+**	92.05	100.00	62.61	99.99
3	Ig mu heavy chain disease protein OS Homo sapiens PE 1 SV 1	MUCB_HUMAN	P04220	4.95	43571	779.9	3	7.67	**+**	**+**	**+**	**+**	87.92	88.14	94.50	99.46
4	Alpha crystallin B chain OS Homo sapiens GN CRYAB PE 1 SV 2	CRYAB_HUMAN	P02511	6.92	20159	3540.02	3	16.57	**+**	**+**	**+**	**-**	88.31	62.61	100.00	0.00
5	Glycogen synthase kinase 3 beta OS Homo sapiens GN GSK3B PE 1 SV 2	GSK3B_HUMAN	P49841	8.89	47258	1194.15	2	5.24	**+**	**+**	**+**	**-**	98.97	99.84	100.00	0.00
6	ATP dependent RNA helicase DDX19A OS Homo sapiens GN DDX19A PE 1 SV 1	DD19A_HUMAN	Q9NUU7	6.16	54431	976.25	3	6.49	**-**	**+**	**+**	**+**	0.00	87.87	62.61	62.61
7	Selenium binding protein 1 OS Homo sapiens GN SELENBP1 PE 1 SV 2	SBP1_HUMAN	Q13228	5.91	52961	693.53	2	6.36	**-**	**-**	**-**	**+**	0.00	0.00	0.00	82.65
8	Ras related protein Rab 15 OS Homo sapiens GN RAB15 PE 1 SV 1	RAB15_HUMAN	P59190	5.39	24676	10042.39	3	2.83	**-**	**-1.3**	**-1.1**	**-**	-85.27	21.25	-11.39	-100.00
9	Serine arginine rich splicing factor 2 OS Homo sapiens GN SRSF2 PE 1 SV 4	SRSF2_HUMAN	Q01130	12.26	25476	5398.974	3	9.05	**-**	**-1**	**-**	**-**	-99.21	-85.06	-99.21	-98.08

10	Ras related protein Rab 12 OS Homo sapiens GN RAB12 PE 1 SV 3	RAB12_HUMAN	Q6IQ22	8.51	27591	5113.237	3	7.38	-	-	-1.5	-	-87.23	-87.23	-61.45	-88.39
11	Actin related protein 2 3 complex subunit 5 OS Homo sapiens GN ARPC5 PE 1 SV 3	ARPC5_HUMAN	O15511	5.31	16377	4083.225	3	12.58	-	-11	-	-13.7	-68.02	-66.04	-68.02	-98.22
12	Seprase OS Homo sapiens GN FAP PE 1 SV 5	SEPR_HUMAN	Q12884	6.2	88397	3632.45	18	3.68	+	-	+	6.6	88.30	0.00	99.99	99.99
13	Secreted frizzled related protein 1 OS Homo sapiens GN SFRP1 PE 1 SV 1	SFRP1_HUMAN	Q8N474	8.97	36298	2288.906	5	6.05	-	-	-	2.1	-85.50	-85.50	-85.50	99.91
14	Vitamin D binding protein OS Homo sapiens GN GC PE 1 SV 1	VTDB_HUMAN	P02774	5.24	54560	1715.75	5	9.28	-	-2.9	-2.4	-	-99.99	-99.91	-99.81	-100.00
15	Glycogenin 1 OS Homo sapiens GN GYG1 PE 1 SV 4	GLYG_HUMAN	P46976	5.13	39726	1578.978	6	13.43	+	+	+	1.4	100.00	99.93	99.95	88.70
16	Neutral amino acid transporter B 0 OS Homo sapiens GN SLC1A5 PE 1 SV 2	AAAT_HUMAN	Q15758	5.18	57055	1560.55	4	10.54	+	+	+	2	100.00	88.06	99.95	99.85
17	Endoglin OS Homo sapiens GN ENG PE 1 SV 2	EGLN_HUMAN	P17813	6.14	71605	1173.463	6	7.14	-	-	+	3.7	0.00	0.00	62.61	99.98
18	Deoxyuridine 5 triphosphate nucleotidohydrolase mitochondrial OS Homo sapiens GN DUT PE 1 SV 4	DUT_HUMAN	P33316	9.64	26848	1120.79	3	11.51	-	1.2	1.2	-	-62.61	86.99	90.62	-99.99
19	Uridine cytidine kinase 2 OS Homo sapiens GN UCK2 PE 1 SV 1	UCK2_HUMAN	Q9BZX2	6.25	29470	1075.959	2	9.58	-	-	-3	-	-62.61	-62.61	-23.44	-62.61
20	Integrin alpha 8 OS Homo sapiens GN ITGA8 PE 1 SV 3	ITA8_HUMAN	P53708	5.23	118728	1065.286	3	5.08	-	-	-	7.2	0.00	0.00	0.00	99.63
21	Septin 14 OS Homo sapiens GN SEPT14 PE 1 SV 2	SEP14_HUMAN	Q6ZU15	5.81	50481	1027.321	2	3.7	+	+	+	-1.9	98.45	87.29	67.95	-73.68
22	Cyclin dependent kinase 1 OS Homo sapiens GN CDK1 PE 1 SV 3	CDK1_HUMAN	P06493	8.76	34153	1024.765	2	2.69	+	+	+	-	99.94	87.79	88.29	-72.59
23	Tricarboxylate transport protein mitochondrial OS Homo sapiens GN SLC25A1 PE 1 SV 2	TXTP_HUMAN	P53007	10.22	34355	935.4251	2	15.11	+	+	+	3.6	99.99	100.00	100.00	99.82

24	Lysophosphatidic acid receptor 1 OS Homo sapiens GN LPAR1 PE 1 SV 3	LPAR1_HUMAN	Q92633	8.49	41737	934.343	3	15.66	-	-	+	**3.5**	0.00	0.00	87.90	99.91
25	Kinesin light chain 1 OS Homo sapiens GN KLC1 PE 1 SV 2	KLC1_HUMAN	Q07866	5.8	65823	909.7861	2	4.54	-	**-1.8**	**-1.1**	**-1.5**	-99.95	-97.31	-71.07	-95.03
26	Interferon induced protein with tetratricopeptide repeats 3 OS Homo sapiens GN IFIT3 PE 1 SV 1	IFIT3_HUMAN	O14879	4.95	56726	900.6319	3	10	+	-	+	**4.5**	88.00	0.00	100.00	99.92
27	Caspase 1 OS Homo sapiens GN CASP1 PE 1 SV 1	CASP1_HUMAN	P29466	5.53	45843	851.947	4	11.14	+	-	+	**2.8**	100.00	0.00	86.83	100.00
28	Metalloproteinase inhibitor 3 OS Homo sapiens GN TIMP3 PE 1 SV 2	TIMP3_HUMAN	P35625	8.76	24829	810.8984	2	10.9	-	-	-	**1**	0.00	0.00	0.00	11.51
29	Integrator complex subunit 4 like protein 2 OS Homo sapiens GN INTS4L2 PE 2 SV 1	IN4L2_HUMAN	Q2T9F4	5.77	49568	792.9083	4	10.02	-	-	**-1.1**	+	-85.25	-85.25	-53.27	62.61
30	Transmembrane glycoprotein NMB OS Homo sapiens GN GPNMB PE 1 SV 2	GPNMB_HUMAN	Q14956	6.17	64664	1467.107	4	6.12	**11.7**	-	**14.8**	**13.7**	99.69	-87.47	99.97	99.53
35	Protein Wnt 5a OS Homo sapiens GN WNT5A PE 1 SV 2	WNT5A_HUMAN	P41221	8.37	43708	7620.229	12	2.89	**4**	-	-	**6.5**	49.68	-62.61	-62.61	100.00
36	Protein S100 A4 OS Homo sapiens GN S100A4 PE 1 SV 1	S10A4_HUMAN	P26447	5.77	11957	11353.28	6	33.66	**2.8**	-	**12.9**	**2.2**	99.09	-62.61	99.98	99.96
37	Glutathione S transferase theta 1 OS Homo sapiens GN GSTT1 PE 1 SV 4	GSTT1_HUMAN	P30711	7.32	27506	965.9648	2	14.58	**2.6**	**1.9**	-	**3.6**	41.06	98.95	-62.61	99.94
38	Cellular retinoic acid binding protein 2 OS Homo sapiens GN CRABP2 PE 1 SV 2	RABP2_HUMAN	P29373	5.18	15864	39530.23	9	15.22	**2.4**	**-1.1**	-	**20.1**	78.86	-6.54	-62.61	99.98
39	Serine arginine rich splicing factor 7 OS Homo sapiens GN SRSF7 PE 1 SV 1	SRSF7_HUMAN	Q16629	12.22	27595	1281.824	3	12.61	**1.2**	**2**	-	**-1.5**	88.04	97.31	-99.99	-94.01
40	Serine arginine rich splicing factor 3 OS Homo sapiens GN SRSF3 PE 1 SV 1	SRSF3_HUMAN	P84103	12.04	19558	1433.102	2	12.8	**1.1**	**1.9**	-	**-1.3**	36.40	95.11	-99.14	-60.36

41	Serine arginine rich splicing factor 6 OS Homo sapiens GN SRSF6 PE 1 SV 2	SRSF6_HUMAN	Q13247	11.82	39701	878.3026	2	4.65	**-1**	**5.2**	**-**	**-2.1**	-7.53	99.94	-99.95	-89.00
42	Hemopexin OS Homo sapiens GN HPX PE 1 SV 2	HEMO_HUMAN	P02790	6.57	52418	7467.361	8	18.83	**-1.3**	**-**	**-**	**19**	-93.55	-99.98	-99.98	100.00
43	Erlin 1 OS Homo sapiens GN ERLIN1 PE 1 SV 1	ERLN1_HUMAN	O75477	7.94	39097	911.3351	2	6.07	**-1.5**	**1.1**	**-**	**-3.8**	-35.65	-42.96	-85.04	-99.77
44	High mobility group protein HMGI C OS Homo sapiens GN HMGA2 PE 1 SV 1	HMGA2_HUMAN	P52926	11.11	11832	7399.867	2	21.1	**-1.5**	**1.1**	**-**	**1**	-79.60	25.48	-99.48	10.78
45	Vasorin OS Homo sapiens GN VASN PE 1 SV 1	VASN_HUMAN	Q6EMK4	7.09	72796	2650.962	5	10.25	**-1.6**	**-**	**-1.2**	**5.8**	57.07	-62.61	-9.73	99.96
46	Collagen alpha 2 I chain OS Homo sapiens GN COL1A2 PE 1 SV 7	CO1A2_HUMAN	P08123	9.22	129827	7426.908	22	2.78	**-1.7**	**-**	**-1.2**	**4.7**	-70.05	-86.79	26.18	100.00
47	Growth differentiation factor 15 OS Homo sapiens GN GDF15 PE 1 SV 3	GDF15_HUMAN	Q99988	9.94	34654	1143.604	2	14.29	**-1.9**	**-**	**-1.9**	**+**	-99.68	-100.00	-99.97	100.00
48	Erlin 2 OS Homo sapiens GN ERLIN2 PE 1 SV 1	ERLN2_HUMAN	O94905	5.32	38068	871.1386	2	2.95	**-2.4**	**-1.5**	**-**	**1.4**	-65.00	-67.34	-87.73	98.83
49	CD109 antigen OS Homo sapiens GN CD109 PE 1 SV 2	CD109_HUMAN	Q6YHK3	5.48	162602	1050.886	12	1.66	**-3.1**	**-**	**-2**	**2.2**	-99.85	-100.00	-99.91	99.99
50	Collagen alpha 1 III chain OS Homo sapiens GN COL3A1 PE 1 SV 4	CO3A1_HUMAN	P02461	6.17	139819	1612.716	11	1.91	**-3.5**	**-**	**-2.7**	**+**	-30.94	-62.61	5.05	99.99
51	Protein phosphatase 1G OS Homo sapiens GN PPM1G PE 1 SV 1	PPM1G_HUMAN	O15355	4.07	59956	981.1996	4	8.42	**-1.1**	**1.5**	**-**	**-1.7**	-41.42	98.13	-99.92	-98.96
52	Prothymosin alpha OS Homo sapiens GN PTMA PE 1 SV 2	PTMA_HUMAN	P06454	3.45	12203	17937.57	3	12.61	**1.7**	**3.4**	**-**	**1.1**	97.33	99.91	-99.91	38.96
53	Tropomodulin 2 OS Homo sapiens GN TMOD2 PE 1 SV 1	TMOD2_HUMAN	Q9NZR1	5.03	39595	996.0465	2	9.97	**10**	**2.6**	**6.8**	**+**	100.00	18.55	99.42	99.39
54	Nicotinate nucleotide pyrophosphorylase carboxylating OS Homo sapiens GN QPRT PE 1 SV 3	NADC_HUMAN	Q15274	5.77	31188	7569.113	6	9.43	**3.4**	**-4**	**3.3**	**+**	99.99	-99.73	99.95	100.00
55	Heat shock related 70 kDa protein 2 OS Homo sapiens GN HSPA2 PE 1 SV 1	HSP72_HUMAN	P54652	5.41	70306	21262.09	18	5.79	**9.2**	**18.8**	**12.4**	**2.8**	100.00	100.00	100.00	99.99

56	ADP ATP translocase 2 OS Homo sapiens GN SLC25A5 PE 1 SV 7	ADT2_HUMAN	P05141	9.99	33080	8690.069	22	11.74	**3.4**	**3.1**	**3.5**	**1.6**	100.00	100.00	100.00	99.87
57	Dipeptidyl peptidase 4 OS Homo sapiens GN DPP4 PE 1 SV 2	DPP4_HUMAN	P27487	5.61	88963	3949.329	25	4.7	**3.3**	**1.7**	**5.6**	**+**	100.00	98.90	99.99	99.95
58	GTP AMP phosphotransferase AK4 mitochondrial OS Homo sapiens GN AK4 PE 1 SV 1	KAD4_HUMAN	P27144	8.69	25382	2702.29	5	4.93	**3**	**6.2**	**2.1**	**+**	99.95	100.00	99.90	0.00
59	Calponin 1 OS Homo sapiens GN CNN1 PE 1 SV 2	CNN1_HUMAN	P51911	9.38	33342	10285	12	24.58	**2.8**	**-1.6**	**-1.3**	**-**	100.00	-99.77	-99.68	-82.80
60	EGF like repeat and discoidin I like domain containing protein 3 OS Homo sapiens GN EDIL3 PE 1 SV 1	EDIL3_HUMAN	O43854	6.99	55134	14036.29	26	7.92	**2.4**	**-3.1**	**1.9**	**-**	100.00	-99.99	100.00	-100.00
61	Aldo keto reductase family 1 member B15 OS Homo sapiens GN AKR1B15 PE 2 SV 1	AK1BF_HUMAN	C9JRZ8	6.01	39631	1700.866	2	6.4	**1.9**	**-108.6**	**-62**	**+**	99.98	-100.00	-100.00	91.91
62	Intercellular adhesion molecule 1 OS Homo sapiens GN ICAM1 PE 1 SV 2	ICAM1_HUMAN	P05362	7.92	58624	6172.111	18	16.73	**1.8**	**-2.8**	**3.5**	**+**	97.76	-99.01	99.99	100.00
63	Retinol dehydrogenase 11 OS Homo sapiens GN RDH11 PE 1 SV 2	RDH11_HUMAN	Q8TC12	9	35785	1810.5	4	10.69	**1.7**	**2.2**	**1.2**	**+**	99.93	99.98	93.87	100.00
64	Coiled coil domain containing protein 57 OS Homo sapiens GN CCDC57 PE 2 SV 2	CCD57_HUMAN	Q2TAC2	6.11	103452	1614.055	6	12.01	**1.7**	**1.4**	**1.5**	**-**	87.38	79.40	88.20	-100.00
65	Serine beta lactamase like protein LACTB mitochondrial OS Homo sapiens GN LACTB PE 1 SV 2	LACTB_HUMAN	P83111	8.64	61150	1331.331	4	6.58	**1.7**	**-1**	**1.2**	**+**	99.73	-11.97	65.32	72.60
66	Connective tissue growth factor OS Homo sapiens GN CTGF PE 1 SV 2	CTGF_HUMAN	P29279	7.86	40316	1452.084	4	14.04	**1.7**	**1.2**	**1.4**	**+**	98.64	85.79	96.63	87.68
67	FAS associated factor 2 OS Homo sapiens GN FAF2 PE 1 SV 2	FAF2_HUMAN	Q96CS3	5.31	52966	880.4904	2	9.89	**1.4**	**1.7**	**1.3**	**+**	86.46	99.83	91.64	99.97
68	Mammalian ependymin related protein 1 OS Homo sapiens GN EPDR1 PE 1 SV 2	EPDR1_HUMAN	Q9UM22	6.42	25893	3062.685	4	14.29	**1.3**	**2.3**	**1.8**	**-**	98.74	99.50	99.57	-62.61

69	OCIA domain containing protein 2 OS Homo sapiens GN OCIAD2 PE 1 SV 1	OCAD2_HUMAN	Q56VL3	9.32	17296	5825.607	5	18.83	**1.3**	**-7.2**	**-1.3**	**+**	98.11	-99.99	-96.81	62.61
70	Tubulointerstitial nephritis antigen like OS Homo sapiens GN TINAGL1 PE 1 SV 1	TINAL_HUMAN	Q9GZM7	6.54	53756	698.4343	4	10.06	**1.2**	**-1.1**	**3.9**	**-**	98.65	-74.06	99.29	0.00
71	Cell surface glycoprotein MUC18 OS Homo sapiens GN MCAM PE 1 SV 2	MUC18_HUMAN	P43121	5.46	72577	1813.984	11	11.76	**1.2**	**-2.1**	**2.1**	**-**	57.80	-99.55	99.97	0.00
72	Creatine kinase B type OS Homo sapiens GN CKB PE 1 SV 1	KCRB_HUMAN	P12277	5.22	42930	5774.735	7	7.35	**1.1**	**8.6**	**1.6**	**-**	74.43	100.00	99.67	-99.95
73	Ras related protein Rab 4B OS Homo sapiens GN RAB4B PE 1 SV 1	RAB4B_HUMAN	P61018	5.7	23872	7937.194	3	17.37	**-1.1**	**1.1**	**2**	**-**	-27.75	36.26	96.25	-99.99
74	Tumor associated calcium signal transducer 2 OS Homo sapiens GN TACSTD2 PE 1 SV 3	TACD2_HUMAN	P09758	8.96	36394	1362.2	2	4.95	**-1.2**	**-1.5**	**1.7**	**-**	78.87	-19.51	98.67	0.00
75	Tubulin folding cofactor B OS Homo sapiens GN TBCB PE 1 SV 2	TBCB_HUMAN	Q99426	4.86	27611	973.4984	2	7.79	**-1.5**	**-1.4**	**-1.4**	**-**	-99.12	-98.73	-99.53	-62.61
76	Keratin type II cytoskeletal 5 OS Homo sapiens GN KRT5 PE 1 SV 3	K2C5_HUMAN	P13647	7.79	62607	1452.75	9	6.1	**17**	**1.9**	**1.7**	**1.4**	100.00	97.31	88.40	92.23
77	HLA class I histocompatibility antigen B 7 alpha chain OS Homo sapiens GN HLA B PE 1 SV 3	1B07_HUMAN	P01889	5.47	40802	22994.44	13	23.48	**11.2**	**-1**	**27.4**	**1.9**	100.00	-1.73	100.00	99.29
78	Keratin type I cytoskeletal 16 OS Homo sapiens GN KRT16 PE 1 SV 4	K1C16_HUMAN	P08779	4.79	51610	1698.697	11	6.34	**7.9**	**-1.2**	**1.5**	**-2.3**	100.00	-96.85	99.13	-99.49
79	Keratin type I cytoskeletal 9 OS Homo sapiens GN KRT9 PE 1 SV 3	K1C9_HUMAN	P35527	4.96	62293	19456.53	23	12.04	**7.8**	**-1.3**	**-1.4**	**-1.3**	100.00	-96.47	-97.88	-99.82
80	Beta actin like protein 2 OS Homo sapiens GN ACTBL2 PE 1 SV 2	ACTBL_HUMAN	Q562R1	5.25	42345	22634.47	16	12.5	**7.3**	**-2.5**	**1.4**	**1.4**	73.41	-92.67	83.32	91.68
81	Keratin type I cytoskeletal 14 OS Homo sapiens GN KRT14 PE 1 SV 4	K1C14_HUMAN	P02533	4.9	51904	1729.375	12	11.44	**6.8**	**-1.5**	**-2.5**	**-1.2**	99.96	-75.84	-90.78	-99.73

82	Phosphate carrier protein mitochondrial OS Homo sapiens GN SLC25A3 PE 1 SV 2	MPCP_HUMAN	Q00325	9.64	40551	6295.737	10	4.42	**6.6**	**5.6**	**2.6**	**1.8**	99.29	99.79	99.96	84.36
83	Farnesyl pyrophosphate synthase OS Homo sapiens GN FDPS PE 1 SV 4	FPPS_HUMAN	P14324	5.76	48789	1568.635	7	2.63	**6.1**	**3.1**	**3.9**	**1.6**	99.91	99.99	98.43	99.56
84	Keratin type II cytoskeletal 1 OS Homo sapiens GN KRT1 PE 1 SV 6	K2C1_HUMAN	P04264	8.27	66210	26970.44	27	4.66	**5.8**	**-1.3**	**-1.4**	**-1.4**	100.00	-92.01	-96.68	-99.07
85	ATP dependent RNA helicase A OS Homo sapiens GN DHX9 PE 1 SV 4	DHX9_HUMAN	Q08211	6.39	142270	719.1813	9	6.22	**5.7**	**6.1**	**3.2**	**-1.7**	100.00	100.00	99.99	-99.95
86	UTP glucose 1 phosphate uridylyltransferase OS Homo sapiens GN UGP2 PE 1 SV 5	UGPA_HUMAN	Q16851	8.31	57111	2389.885	14	6.5	**5.3**	**1.8**	**3.1**	**-1.4**	100.00	95.68	99.21	-98.47
87	Proteolipid protein 2 OS Homo sapiens GN PLP2 PE 1 SV 1	PLP2_HUMAN	Q04941	7.01	17033	6455.277	4	8.55	**5.3**	**2.6**	**5.6**	**1.1**	99.98	99.69	99.99	63.76
88	Mitogen activated protein kinase 3 OS Homo sapiens GN MAPK3 PE 1 SV 4	MK03_HUMAN	P27361	6.29	43478	1695.877	12	7.12	**5.2**	**-1.4**	**2.2**	**1.1**	99.99	-74.87	99.72	21.12
89	Organic solute transporter subunit beta OS Homo sapiens GN SLC51B PE 2 SV 2	OSTB_HUMAN	Q86UW2	4.38	14346	2135.313	3	34.38	**5.2**	**2.4**	**4.6**	**-1.1**	96.19	99.13	99.06	-10.36
90	Transmembrane emp24 domain containing protein 7 OS Homo sapiens GN TMED7 PE 1 SV 2	TMED7_HUMAN	Q9Y3B3	6.49	25514	2811.043	4	10.27	**5.1**	**10**	**6.5**	**1.4**	99.97	99.88	99.98	78.12
91	Ras related protein Rap 1A OS Homo sapiens GN RAP1A PE 1 SV 1	RAP1A_HUMAN	P62834	6.53	21329	2954.511	5	6.52	**4.4**	**1.6**	**6.9**	**6.2**	99.65	99.79	100.00	99.92
92	Glutathione S transferase Mu 2 OS Homo sapiens GN GSTM2 PE 1 SV 2	GSTM2_HUMAN	P28161	5.95	25916	3363.97	9	27.52	**4.4**	**9.8**	**-1.9**	**1.2**	99.99	99.89	-97.42	65.31
93	Peptidyl prolyl cis trans isomerase A like 4G OS Homo sapiens GN PPIAL4G PE 2 SV 1	PAL4G_HUMAN	A2BFH1	9.44	18394	2940.364	2	20.73	**3.3**	**-6.6**	**4.3**	**3.8**	99.99	-99.52	98.31	92.66

94	Guanine nucleotide binding protein G s subunit alpha isoforms XLas OS Homo sapiens GN GNAS PE 1 SV	GNAS1_HUMAN	Q5JWF2	4.72	111766	2894.66	11	4.82	**3.1**	**2.4**	**1.7**	**-7.3**	99.87	98.42	97.16	-99.82
95	Leukocyte elastase inhibitor OS Homo sapiens GN SERPINB1 PE 1 SV 1	ILEU_HUMAN	P30740	5.85	42856	1372.522	4	6.6	**3**	**-1.1**	**5.2**	**2.7**	97.62	-9.22	99.87	99.82
96	Lactadherin OS Homo sapiens GN MFGE8 PE 1 SV 2	MFGM_HUMAN	Q08431	8.06	43921	9587.251	21	12.92	**2.7**	**-3.3**	**6**	**3**	99.91	-99.98	100.00	100.00
97	Pyridoxal kinase OS Homo sapiens GN PDXK PE 1 SV 1	PDXK_HUMAN	O00764	5.7	35330	10159.11	11	19.87	**2.7**	**5.2**	**3.9**	**2.3**	99.99	99.99	100.00	99.99
98	Glutamyl aminopeptidase OS Homo sapiens GN ENPEP PE 1 SV 3	AMPE_HUMAN	Q07075	5.15	109758	1291.259	14	3.66	**2.3**	**7.9**	**3**	**-1.7**	99.90	100.00	99.97	-46.00
99	Membrane associated progesterone receptor component 1 OS Homo sapiens GN PGRMC1 PE 1 SV 3	PGRC1_HUMAN	O00264	4.34	21785	11208.57	4	14.36	**2.3**	**3.2**	**1.1**	**10.1**	98.12	99.69	-29.95	99.98
100	Peptidyl prolyl cis trans isomerase A like 4D OS Homo sapiens GN PPIAL4D PE 3 SV 1	PAL4D_HUMAN	F5H284	9.76	18395	23780.8	4	12.8	**2.3**	**1.9**	**1.5**	**-25.5**	99.87	99.86	88.08	-96.01
101	Heterogeneous nuclear ribonucleoprotein C like 1 OS Homo sapiens GN HNRNPCL1 PE 1 SV 1	HNRCL_HUMAN	O60812	4.74	32199	2749.721	4	15.36	**1.9**	**1.5**	**9.3**	**1.3**	99.42	86.64	100.00	94.61
102	Fumarylacetoacetate hydrolase domain containing protein 2A OS Homo sapiens GN FAHD2A PE 1 SV 1	FAH2A_HUMAN	Q96GK7	8.28	34938	1646.508	8	24.84	**1.9**	**1.2**	**-1.1**	**-20.7**	98.92	48.66	-20.43	-87.75
103	Tryptophan tRNA ligase cytoplasmic OS Homo sapiens GN WARS PE 1 SV 2	SYWC_HUMAN	P23381	5.78	53508	27537.11	26	9.13	**1.8**	**-2.4**	**1.2**	**11.9**	100.00	-99.99	97.66	99.99
104	GTP binding protein SAR1b OS Homo sapiens GN SAR1B PE 1 SV 1	SAR1B_HUMAN	Q9Y6B6	5.69	22524	1850.109	5	23.23	**1.6**	**2.2**	**-3.7**	**-6.6**	96.20	99.91	-99.64	-99.64
105	Delta 3 5 Delta 2 4 dienoyl CoA isomerase mitochondrial OS Homo sapiens GN ECH1 PE 1 SV 2	ECH1_HUMAN	Q13011	8	36158	1373.085	8	11.59	**1.6**	**1.8**	**1.8**	**5**	99.86	99.99	99.97	99.99

106	HLA class I histocompatibility antigen B 41 alpha chain OS Homo sapiens GN HLA B PE 1 SV 1	1B41_HUMAN	P30479	6.08	40881	25085.64	15	25.41	**1.5**	**-1.5**	**3.5**	**10.6**	99.99	-82.18	99.99	100.00
107	Putative heat shock protein HSP 90 beta 3 OS Homo sapiens GN HSP90AB3P PE 5 SV 1	H90B3_HUMAN	Q58FF7	4.51	68667	20672.39	20	6.03	**1.4**	**10.6**	**-1.9**	**9.9**	93.60	100.00	-99.96	99.42
108	High mobility group protein B2 OS Homo sapiens GN HMGB2 PE 1 SV 2	HMGB2_HUMAN	P26583	7.94	24205	6490.066	9	2.87	**1.4**	**5.7**	**2**	**-1.2**	95.89	99.86	68.15	-94.42
109	Serine hydroxymethyltransferase cytosolic OS Homo sapiens GN SHMT1 PE 1 SV 1	GLYC_HUMAN	P34896	7.53	53653	944.2294	2	10.77	**1.3**	**-1.1**	**1.2**	**10**	84.28	-60.11	92.06	100.00
110	HLA class I histocompatibility antigen B 73 alpha chain OS Homo sapiens GN HLA B PE 1 SV 1	1B73_HUMAN	Q31612	5.8	40834	19057.28	11	24.79	**1.3**	**-7.7**	**3.1**	**4.5**	98.00	-99.95	100.00	100.00
111	Erythrocyte band 7 integral membrane protein OS Homo sapiens GN STOM PE 1 SV 3	STOM_HUMAN	P27105	7.99	31902	14084.52	7	12.5	**1.3**	**-1.3**	**1.5**	**15**	84.44	20.14	84.71	100.00
112	Activated RNA polymerase II transcriptional coactivator p15 OS Homo sapiens GN SUB1 PE 1 SV 3	TCP4_HUMAN	P53999	10.07	14395	5232.502	4	8.66	**1.3**	**1.5**	**-8.8**	**-1**	41.39	96.22	-99.60	-8.90
113	Inactive tyrosine protein kinase 7 OS Homo sapiens GN PTK7 PE 1 SV 2	PTK7_HUMAN	Q13308	6.66	119875	3838.873	18	5.14	**1.2**	**1.1**	**1.1**	**15.6**	99.70	81.26	85.83	99.99
114	Core histone macro H2A 1 OS Homo sapiens GN H2AFY PE 1 SV 4	H2AY_HUMAN	O75367	10.23	39788	2871.756	9	15.59	**1.2**	**2.3**	**1.4**	**5.4**	99.89	99.92	99.84	100.00
115	Phosphoserine aminotransferase OS Homo sapiens GN PSAT1 PE 1 SV 2	SERC_HUMAN	Q9Y617	7.59	40822	12543.34	23	8.38	**1.2**	**1.2**	**-1.3**	**15.5**	98.70	89.77	-98.92	100.00
116	Guanine nucleotide binding protein G s subunit alpha isoforms short OS Homo sapiens GN GNAS PE 1 SV	GNAS2_HUMAN	P63092	5.47	46121	2821.014	9	6.6	**1.2**	**1.2**	**1.8**	**5.1**	77.17	79.21	94.61	100.00

117	CD82 antigen OS Homo sapiens GN CD82 PE 1 SV 1	CD82_HUMAN	P27701	4.98	30253	2024.045	5	17.23	**1.2**	**-6.1**	**1.1**	**-6.8**	66.60	-99.31	21.26	-99.54
118	Deoxynucleoside triphosphate triphosphohydrolase SAMHD1 OS Homo sapiens GN SAMHD1 PE 1 SV 2	SAMH1_HUMAN	Q9Y3Z3	6.71	72942	3509.442	13	6.71	**1.2**	**-1**	**3.5**	**10.1**	75.69	-36.62	100.00	100.00
119	Serine arginine rich splicing factor 1 OS Homo sapiens GN SRSF1 PE 1 SV 2	SRSF1_HUMAN	Q07955	10.6	27859	4283.672	13	11.29	**1.1**	**1.9**	**-16.2**	**1.2**	82.81	99.98	-99.99	91.75
120	Elongation factor 1 alpha 2 OS Homo sapiens GN EEF1A2 PE 1 SV 1	EF1A2_HUMAN	Q05639	9.35	50812	17340.53	19	10.37	**1.1**	**-35.2**	**-4.2**	**-1.5**	26.79	-99.84	-99.51	-100.00
121	CD63 antigen OS Homo sapiens GN CD63 PE 1 SV 2	CD63_HUMAN	P08962	7.72	26492	2782.077	2	2.52	**1.1**	**-8**	**1.2**	**-1.7**	59.35	-100.00	98.40	-13.81
122	Aflatoxin B1 aldehyde reductase member 4 OS Homo sapiens GN AKR7L PE 2 SV 6	ARK74_HUMAN	Q8NHP1	6.28	37213	1193.045	2	11.18	**1**	**1.5**	**-5.9**	**2.2**	-56.66	97.41	-99.70	99.88
123	Nucleolin OS Homo sapiens GN NCL PE 1 SV 3	NUCL_HUMAN	P19338	4.4	76672	4076.04	20	2.11	**1**	**1.4**	**-6.1**	**-1.2**	4.45	99.31	-99.99	-91.52
124	RNA binding motif protein X linked like 3 OS Homo sapiens GN RBMXL3 PE 2 SV 2	RMXL3_HUMAN	Q8N7X1	9.15	115793	1049.455	2	2.72	**-1.1**	**1.7**	**8.2**	**2**	-56.10	81.45	99.96	99.59
125	Polypyrimidine tract binding protein 2 OS Homo sapiens GN PTBP2 PE 1 SV 1	PTBP2_HUMAN	Q9UKA9	8.98	57605	702.1445	2	6.97	**-1.1**	**1.4**	**1.1**	**-15.6**	82.61	96.86	92.93	-86.65
126	Protein tweety homolog 3 OS Homo sapiens GN TTYH3 PE 1 SV 3	TTYH3_HUMAN	Q9C0H2	5.04	58514	3665.679	8	4.97	**-1.2**	**-11.3**	**-1.8**	**-1.3**	-66.74	-99.89	-98.35	-67.82
127	Neutral cholesterol ester hydrolase 1 OS Homo sapiens GN NCEH1 PE 1 SV 3	NCEH1_HUMAN	Q6PIU2	6.85	46093	1285.842	5	11.76	**-1.2**	**-5.7**	**-2**	**-1.2**	-98.94	-100.00	-99.99	-97.18
128	Thymidine phosphorylase OS Homo sapiens GN TYMP PE 1 SV 2	TYPH_HUMAN	P19971	5.21	50355	1853.895	7	3.11	**-1.2**	**1.1**	**8.6**	**3.2**	-30.15	14.67	100.00	99.99
129	Catalase OS Homo sapiens GN CAT PE 1 SV 3	CATA_HUMAN	P04040	6.97	59984	7442.282	21	7.02	**-1.2**	**5.4**	**1.2**	**1.2**	-97.60	100.00	77.50	88.07
130	14 3 3 protein sigma OS Homo sapiens GN SFN PE 1 SV 1	1433S_HUMAN	P31947	4.48	27888	6397.539	5	4.03	**-1.2**	**-1.1**	**-69.3**	**-1.1**	-99.09	-92.89	-100.00	-38.60

131	Putative tubulin beta chain like protein ENSP00000290377 OS Homo sapiens PE 5 SV 2	YI016_HUMAN	A6NKZ8	4.57	42231	13847.46	9	10.48	**-1.2**	**-43.4**	**-1.2**	**-29.6**	-97.50	-100.00	-98.13	-99.96
132	HLA class I histocompatibility antigen A 68 alpha chain OS Homo sapiens GN HLA A PE 1 SV 4	1A68_HUMAN	P01891	6.23	41194	15599.33	13	24.66	**-1.3**	**-5.2**	**-2.5**	**5.2**	-96.30	-99.96	-99.84	99.10
133	CD9 antigen OS Homo sapiens GN CD9 PE 1 SV 4	CD9_HUMAN	P21926	6.9	25986	19258	5	21.05	**-1.3**	**4.6**	**6.4**	**-1.2**	-99.15	99.61	100.00	-78.96
134	UDP N acetylhexosamine pyrophosphorylase OS Homo sapiens GN UAP1 PE 1 SV 3	UAP1_HUMAN	Q16222	5.89	59168	3515.12	9	8.24	**-1.6**	**-4.9**	**-2.3**	**-7**	-98.39	-100.00	-99.76	-100.00
135	Myosin light chain 6B OS Homo sapiens GN MYL6B PE 1 SV 1	MYL6B_HUMAN	P14649	5.42	22878	31199.26	3	13.46	**-1.8**	**1.2**	**-1.4**	**211**	-97.39	74.45	-87.68	99.99
136	Serine threonine protein phosphatase 5 OS Homo sapiens GN PPP5C PE 1 SV 1	PPP5_HUMAN	P53041	5.84	57449	890.6174	5	4.81	**-1.9**	**1**	**-1.1**	**-1.1**	-99.34	7.98	-28.62	-43.65
137	High mobility group protein B1 OS Homo sapiens GN HMGB1 PE 1 SV 3	HMGB1_HUMAN	P09429	5.45	25065	10294.76	8	2.79	**-1.9**	**1.2**	**-10.5**	**-1.1**	-99.54	98.44	-99.99	-50.29
138	POTE ankyrin domain family member E OS Homo sapiens GN POTEE PE 1 SV 3	POTEE_HUMAN	Q6S8J3	5.77	122961	30739.02	25	8.84	**-2.1**	**37.9**	**51.9**	**28.4**	-99.82	100.00	100.00	100.00
139	Alpha 2 macroglobulin OS Homo sapiens GN A2M PE 1 SV 3	A2MG_HUMAN	P01023	6	164717	1575.234	12	4.82	**-2.3**	**-3.8**	**-2.7**	**-56.8**	-99.98	-99.99	-99.98	-100.00
140	Acetyl CoA acetyltransferase mitochondrial OS Homo sapiens GN ACAT1 PE 1 SV 1	THIL_HUMAN	P24752	9.12	45485	5095.615	17	21.08	**-2.8**	**-1.5**	**-1.2**	**-5.3**	-96.09	-64.05	-36.42	-93.65
141	C 1 tetrahydrofolate synthase cytoplasmic OS Homo sapiens GN MTHFD1 PE 1 SV 3	C1TC_HUMAN	P11586	6.91	102244	1495.12	24	2.78	**-2.9**	**-1.1**	**-1.1**	**-6**	-61.40	-6.57	-5.97	-99.92
142	HLA class I histocompatibility antigen B 13 alpha chain OS Homo sapiens GN HLA B PE 2 SV 1	1B13_HUMAN	P30461	5.72	40817	14769.7	9	22.38	**-2.9**	**-3.8**	**-6.9**	**16.3**	-99.97	-99.88	-99.99	99.95

143	60S ribosomal protein L26 OS Homo sapiens GN RPL26 PE 1 SV 1	RL26_HUMAN	P61254	10.98	17258	3027.559	3	10.34	**-3**	**1.1**	**-5.2**	**1.6**	-99.99	51.08	-99.99	97.90
144	40S ribosomal protein S14 OS Homo sapiens GN RPS14 PE 1 SV 3	RS14_HUMAN	P62263	10.53	16444	15083.68	11	31.13	**-3**	**-2.1**	**-5.4**	**-1.5**	-99.96	-99.90	-99.99	-99.79
145	Transforming growth factor beta induced protein ig h3 OS Homo sapiens GN TGFBI PE 1 SV 1	BGH3_HUMAN	Q15582	7.52	75308	10586.17	17	9.81	**-3.1**	**-3.8**	**-6.1**	**16.3**	-99.98	-99.99	-100.00	100.00
146	HLA class I histocompatibility antigen Cw 16 alpha chain OS Homo sapiens GN HLA C PE 2 SV 1	1C16_HUMAN	Q29960	6.08	41266	21197.58	11	25.96	**-3.1**	**-3**	**-1.6**	**5.5**	-87.28	-86.01	-65.92	99.99
147	Coatomer subunit beta OS Homo sapiens GN COPB2 PE 1 SV 2	COPB2_HUMAN	P35606	4.97	103343	3793.894	43	8.94	**-3.6**	**1.2**	**-1.2**	**-5.6**	-65.68	24.10	-16.57	-100.00
148	Serotransferrin OS Homo sapiens GN TF PE 1 SV 3	TRFE_HUMAN	P02787	6.75	79345	65982.49	48	7.45	**-3.7**	**-3.4**	**-2.6**	**9**	-100.00	-100.00	-100.00	100.00
149	Eukaryotic translation initiation factor 5A 2 OS Homo sapiens GN EIF5A2 PE 1 SV 3	IF5A2_HUMAN	Q9GZV4	5.24	17135	7698.182	8	41.83	**-3.7**	**-1.5**	**-2.7**	**-5.3**	-99.98	-99.32	-99.97	-99.99
150	60S ribosomal protein L5 OS Homo sapiens GN RPL5 PE 1 SV 3	RL5_HUMAN	P46777	10.02	34591	6575.533	10	13.8	**-4.2**	**-1**	**-8.1**	**-1.4**	-99.97	-26.98	-99.98	-96.27
151	Protein CYR61 OS Homo sapiens GN CYR61 PE 1 SV 1	CYR61_HUMAN	O00622	8.09	44194	2089.063	5	10.24	**-4.6**	**-2.3**	**-5.9**	**-2.6**	-99.99	-99.97	-99.99	-100.00
152	Epoxide hydrolase 1 OS Homo sapiens GN EPHX1 PE 1 SV 1	HYEP_HUMAN	P07099	6.84	53177	3893.229	18	3.96	**-4.7**	**-2.2**	**-2.2**	**-7**	-100.00	-100.00	-100.00	-99.99
153	Valine tRNA ligase OS Homo sapiens GN VARS PE 1 SV 4	SYVC_HUMAN	P26640	7.36	141731	1580.284	33	3.48	**-5**	**-2.1**	**-2.9**	**-1.2**	-99.99	-99.96	-99.98	-93.03
154	NAD P H dehydrogenase quinone 1 OS Homo sapiens GN NQO1 PE 1 SV 1	NQO1_HUMAN	P15559	9.26	30925	18933.26	11	16.06	**-5.1**	**-9.1**	**-4.3**	**1.5**	-100.00	-100.00	-100.00	99.19
155	Carbonyl reductase NADPH 3 OS Homo sapiens GN CBR3 PE 1 SV 3	CBR3_HUMAN	O75828	5.75	31249	6834.446	9	8.66	**-5.1**	**1.7**	**-1.4**	**2**	-90.67	82.22	-43.60	98.77
156	Histone H4 OS Homo sapiens GN HIST1H4A PE 1 SV 2	H4_HUMAN	P62805	11.77	11367	78191.55	10	17.48	**-5.2**	**1.1**	**-1.2**	**1.6**	-100.00	81.06	-98.77	100.00

157	Integrin alpha 2 OS Homo sapiens GN ITGA2 PE 1 SV 1	ITA2_HUMAN	P17301	4.99	130550	1191.372	10	4.57	**-5.2**	**1.3**	**1.4**	**1**	-95.40	48.61	51.89	42.67
158	Hemoglobin subunit epsilon OS Homo sapiens GN HBE1 PE 1 SV 2	HBE_HUMAN	P02100	9.18	16260	8652.665	3	8.16	**-5.3**	**-1.4**	**-1.6**	**-6.1**	-99.78	-94.28	-80.50	-99.98
159	Guanine nucleotide binding protein G t subunit alpha 2 OS	GNAT2_HUMAN	P19087	4.93	40746	2288.561	5	7.34	**-5.6**	**-2**	**-1.3**	**-4.4**	-99.94	-94.64	-94.17	-96.19
160	40S ribosomal protein S25 OS Homo sapiens GN RPS25 PE 1 SV 1	RS25_HUMAN	P62851	10.58	13799	12280.78	4	21.6	**-5.9**	**-2.8**	**-5.3**	**-1.1**	-99.46	-98.43	-99.44	-64.09
161	Histone H2A x OS Homo sapiens GN H2AFX PE 1 SV 2	H2AX_HUMAN	P16104	11.16	15145	60892.66	6	11.89	**-6.2**	**1.2**	**-1.7**	**2**	-100.00	98.15	-99.99	99.89
162	Niban like protein 1 OS Homo sapiens GN FAM129B PE 1 SV 3	NIBL1_HUMAN	Q96TA1	5.76	84651	3471.417	27	2.68	**-6.7**	**-2.4**	**-1.7**	**-6.6**	-100.00	-100.00	-99.91	-100.00
163	Hemoglobin subunit alpha OS Homo sapiens GN HBA1 PE 1 SV 2	HBA_HUMAN	P69905	9.18	15315	21076.16	6	8.45	**-6.9**	**2.1**	**1.4**	**1**	-100.00	99.84	99.97	88.01
164	V type proton ATPase subunit F OS Homo sapiens GN ATP6V1F PE 1 SV 2	VATF_HUMAN	Q16864	5.15	13370	2184.358	2	15.97	**-7**	**-3.2**	**-2.2**	**-13.1**	-99.98	-99.96	-99.87	-99.62
165	Lactotransferrin OS Homo sapiens GN LTF PE 1 SV 6	TRFL_HUMAN	P02788	8.01	80064	3377.094	14	14.37	**-7**	**-2.9**	**-2.7**	**-14.7**	-100.00	-100.00	-100.00	-100.00
166	60S ribosomal protein L11 OS Homo sapiens GN RPL11 PE 1 SV 2	RL11_HUMAN	P62913	9.95	20481	5629.544	5	8.99	**-7.3**	**-3.4**	**-7.4**	**-1.3**	-100.00	-100.00	-100.00	-97.37
167	V type proton ATPase subunit B kidney isoform OS Homo sapiens GN ATP6V1B1 PE 1 SV 3	VATB1_HUMAN	P15313	5.31	57232	1084.387	9	12.28	**-7.5**	**1.2**	**-5.1**	**1.8**	-99.81	65.17	-99.73	99.98
168	Interferon induced GTP binding protein Mx2 OS Homo sapiens GN MX2 PE 1 SV 1	MX2_HUMAN	P20592	8.99	82545	1456.002	6	8.25	**-7.5**	**-3.6**	**-1.9**	**1.4**	-43.37	-48.09	40.49	89.01
169	Nucleoside diphosphate kinase A OS Homo sapiens GN NME1 PE 1 SV 1	NDKA_HUMAN	P15531	5.77	17320	24311.76	6	42.11	**-7.9**	**-1.3**	**6.1**	**-1.2**	-99.97	-88.88	99.99	-99.29
170	Histone H3 2 OS Homo sapiens GN HIST2H3A PE 1 SV 3	H32_HUMAN	Q71DI3	11.68	15445	44556.89	8	4.41	**-7.9**	**-1.3**	**-1.9**	**1.2**	-99.99	-98.54	-99.92	83.83
171	ATP dependent RNA helicase DDX39A OS Homo sapiens GN DDX39A PE 1 SV 2	DX39A_HUMAN	O00148	5.33	49643	5421.293	20	24.36	**-8.3**	**-2.1**	**-1.3**	**-4.1**	-100.00	-99.97	-99.67	-100.00

172	Putative elongation factor 1 alpha like 3 OS Homo sapiens GN EEF1A1P5 PE 5 SV 1	EF1A3_HUMAN	Q5VTE0	9.41	50527	34437.56	20	19.26	**-8.7**	**-1.1**	**-1.2**	**-1.2**	-100.00	-76.84	-99.66	-97.60
173	Tubulin beta 8 chain like protein LOC260334 OS Homo sapiens PE 1 SV 1	TBB8L_HUMAN	A6NNZ2	4.56	50200	27595.83	10	11.71	**-9.2**	**-1.2**	**-12**	**-5.4**	-100.00	-96.05	-100.00	-99.44
174	60S ribosomal protein L12 OS Homo sapiens GN RPL12 PE 1 SV 1	RL12_HUMAN	P30050	9.9	17990	18720.47	7	30.3	**-9.8**	**-9.6**	**-7.9**	**-1**	-99.95	-99.95	-99.94	-1.57
175	Hemoglobin subunit beta OS Homo sapiens GN HBB PE 1 SV 2	HBB_HUMAN	P68871	6.88	16112	8194.478	3	6.12	**-11.5**	**1.4**	**-1.1**	**-2.1**	-99.81	80.03	-74.21	-97.82
176	HLA class I histocompatibility antigen A 3 alpha chain OS Homo sapiens GN HLA A PE 1 SV 2	1A03_HUMAN	P04439	5.58	41126	23487.08	16	27.67	**-13.8**	**-9.5**	**-21.2**	**3.1**	-100.00	-100.00	-100.00	99.98
177	Keratin type II cuticular Hb5 OS Homo sapiens GN KRT85 PE 1 SV 1	KRT85_HUMAN	P78386	6.2	57342	1344.757	8	6.31	**-14.6**	**-17.4**	**-6.1**	**-3.7**	-99.39	-99.42	-99.11	-98.50
178	Inter alpha trypsin inhibitor heavy chain H2 OS Homo sapiens GN ITIH2 PE 1 SV 2	ITIH2_HUMAN	P19823	6.4	106920	948.4495	10	6.03	**-14.6**	**-3.9**	**-2.5**	**-7.2**	-99.84	-99.60	-98.78	-100.00
179	3 hydroxyisobut+te dehydrogenase mitochondrial OS Homo sapiens GN HIBADH PE 1 SV 2	3HIDH_HUMAN	P31937	8.15	35728	2318.316	15	18.45	**-18.3**	**1.1**	**1.2**	**-3.8**	-99.65	50.48	76.36	-82.03
180	Histone H2B type 1 A OS Homo sapiens GN HIST1H2BA PE 1 SV 3	H2B1A_HUMAN	Q96A08	10.74	14168	4066.84	3	18.9	**-19.4**	**-2.7**	**-1.6**	**-2.7**	-99.97	-99.84	-97.84	-99.91
181	Keratin type II cuticular Hb4 OS Homo sapiens GN KRT84 PE 2 SV 2	KRT84_HUMAN	Q9NSB2	7.42	65983	2680.325	12	9.83	**-22.8**	**-2.4**	**-2.5**	**-21.2**	-100.00	-100.00	-100.00	-100.00
182	Tubulin alpha 8 chain OS Homo sapiens GN TUBA8 PE 1 SV 1	TBA8_HUMAN	Q9NY65	4.76	50778	55144.62	16	10.47	**-22.9**	**-4.9**	**-3.6**	**-31.9**	-100.00	-100.00	-100.00	-100.00
183	HLA class I histocompatibility antigen Cw 17 alpha chain OS Homo sapiens GN HLA C PE 1 SV 1	1C17_HUMAN	Q95604	6.34	41637	5709.015	6	14.52	**-26.7**	**-1.6**	**2.8**	**-1.5**	-99.93	-87.26	99.75	-62.62
184	Alpha fetoprotein OS Homo sapiens GN AFP PE 1 SV 1	FETA_HUMAN	P02771	5.36	70503	1749.228	10	4.6	**-29.1**	**-3.1**	**-1.7**	**-47.9**	-100.00	-100.00	-99.99	-100.00
185	Putative heat shock protein HSP 90 alpha A2 OS Homo sapiens GN HSP90AA2 PE 1 SV 2	HS902_HUMAN	Q14568	4.38	39479	9550.752	11	6.71	**-31.1**	**-11.9**	**-1.2**	**-1.7**	-98.46	-98.14	-45.58	-99.84

186	DNA J homolog subfamily A member 2 OS Homo sapiens GN DNAJA2 PE 1 SV 1	DNJA2_HUMAN	O60884	6.03	46373	1355.59	4	6.07	**-32.4**	**-2.1**	**-2**	**-6.8**	-99.99	-99.84	-99.83	-95.11
187	Vitronectin OS Homo sapiens GN VTN PE 1 SV 1	VTNC_HUMAN	P04004	5.43	55104	4185.004	7	20.08	**-44.7**	**-2**	**-6.2**	**-1.7**	-99.99	-96.81	-99.91	-99.62
188	60S ribosomal protein L14 OS Homo sapiens GN RPL14 PE 1 SV 4	RL14_HUMAN	P50914	11.4	23546	4625.685	7	12.56	**-45**	**-40.6**	**-23.4**	**-1.1**	-99.72	-99.72	-99.70	-64.33
189	N 6 adenine specific DNA methyltransferase 2 OS Homo sapiens GN N6AMT2 PE 2 SV 1	N6MT2_HUMAN	Q8WVE0	4.27	24905	1514.131	2	16.36	**-45.9**	**-1.1**	**-1**	**-4.3**	-99.65	-22.87	-5.68	-98.88
190	Fumarylacetoacetate hydrolase domain containing protein 2B OS Homo sapiens GN FAHD2B PE 2 SV 1	FAH2B_HUMAN	Q6P2I3	7.68	34955	1737.008	5	29.62	**-58**	**-1.8**	**-1.7**	**-4.6**	-99.84	-95.44	-95.44	-76.52
191	Alpha 2 HS glycoprotein OS Homo sapiens GN AHSG PE 1 SV 1	FETUA_HUMAN	P02765	5.33	40123	11011.86	11	27.79	**-81.1**	**-1.8**	**-1.5**	**-8.2**	-99.99	-99.81	-99.50	-100.00
192	Nestin OS Homo sapiens GN NES PE 1 SV 2	NEST_HUMAN	P48681	4.15	177895	1313.037	34	12.71	**-1.3**	**-1.4**	**-1.1**	**-1.5**	-98.08	-99.15	-82.59	-99.82
193	Adipocyte plasma membrane associated protein OS Homo sapiens GN APMAP PE 1 SV 2	APMAP_HUMAN	Q9HDC9	5.75	46652	3356.65	19	28.13	**1**	**1.3**	**-1.2**	**-1.3**	5.06	94.43	-69.42	-99.11
194	Myosin light chain kinase smooth muscle OS Homo sapiens GN MYLK PE 1 SV 4	MYLK_HUMAN	Q15746	5.77	213453	2146.201	19	2.4	**1.7**	**4.8**	**1.3**	**2.6**	98.31	99.99	75.21	98.98
195	Myosin 10 OS Homo sapiens GN MYH10 PE 1 SV 3	MYH10_HUMAN	P35580	5.27	229969	3029.458	56	4.4	**1.7**	**1.4**	**1.2**	**-1.1**	99.79	98.29	93.04	-34.16
196	Myosin regulatory light chain 12A OS Homo sapiens GN MYL12A PE 1 SV 2	ML12A_HUMAN	P19105	4.45	19851	50248.41	11	44.44	**1.6**	**1**	**1.2**	**1**	99.96	34.30	98.22	90.47
197	Myosin regulatory light chain 12B OS Homo sapiens GN MYL12B PE 1 SV 2	ML12B_HUMAN	O14950	4.49	19836	48107.02	11	44.19	**1.3**	**1.4**	**1.4**	**2.1**	99.68	99.91	99.91	100.00
198	Tropomyosin alpha 1 chain OS Homo sapiens GN TPM1 PE 1 SV 2	TPM1_HUMAN	P09493	4.49	32766	17023.25	10	5.63	**1.6**	**1.4**	**1.6**	**-1.1**	99.98	99.98	100.00	-88.57
199	Tropomyosin alpha 4 chain OS Homo sapiens GN TPM4 PE 1 SV 3	TPM4_HUMAN	P67936	4.47	28636	18189.9	14	18.95	**1.9**	**1.8**	**2.1**	**1.1**	99.91	99.92	99.92	88.52

200	Desmin OS Homo sapiens GN DES PE 1 SV 3	DESM_HUMAN	P17661	5.03	53593	5545.81	13	5.53	**3.3**	**1.9**	**2.6**	**1.4**	99.30	95.84	92.37	84.06
201	Caldesmon OS Homo sapiens GN CALD1 PE 1 SV 3	CALD1_HUMAN	Q05682	5.44	93288	15523.95	23	4.29	**1.2**	**-1.1**	**-1.6**	**1.3**	88.84	-92.16	-99.47	99.54
202	Peroxisomal multifunctional enzyme type 2 OS Homo sapiens GN HSD17B4 PE 1 SV 3	DHB4_HUMAN	P51659	9.08	80143	2980.72	24	7.34	**1.2**	**1.7**	**1**	**1.4**	67.30	98.09	2.28	89.36
203	Tubulin beta 3 chain OS Homo sapiens GN TUBB3 PE 1 SV 2	TBB3_HUMAN	Q13509	4.64	50889	51656.5	26	12.89	**-1.1**	**-1**	**1.1**	**1.2**	-92.37	-33.07	68.12	91.11
204	Alpha enolase OS Homo sapiens GN ENO1 PE 1 SV 2	ENOA_HUMAN	P06733	7.17	47511	98940.22	29	21.66	**-1**	**1.3**	**1**	**1**	-16.35	97.56	72.52	30.41
205	Vimentin OS Homo sapiens GN VIM PE 1 SV 4	VIME_HUMAN	P08670	4.86	53709	102289.6	39	3.43	**-1.5**	**-1.1**	**-1.2**	**1**	-99.99	-99.83	-99.55	13.59
206	Nucleoside diohosphate kinase A OS Homo sapiens GN NME1 PE 1 SV 1	NDKA_HUMAN	P15531	5.77	17320	24311.76	6	42.11	**-7.9**	**-1.3**	**6.1**	**-1.2**	-99.97	-88.88	99.99	-99.29
207	Cadherin 13 OS Homo sapiens GN CDH13 PE 1 SV 1	CAD13_HUMAN	P55290	4.61	78743	3344.156	12	3.37	**-1.3**	**-3.7**	**-4.1**	**1.5**	-95.73	-99.91	-99.92	91.88
208	Catenin alpha 2 OS Homo sapiens GN CTNNA2 PE 1 SV 5	CTNA2_HUMAN	P26232	5.37	106112	1818.535	7	2.83	**-1.3**	**1.2**	**1.1**	**1.6**	-71.89	60.03	52.41	96.81
209	Glutathione S transferase P OS Homo sapiens GN GSTP1 PE1 SV 2	GSTP1_HUMAN	P09211	5.28	23584	81527.2	12	8.57	**1.5**	**-1.2**	**1.2**	**1.6**	99.96	-99.06	98.94	100.00
210	Keratin type II cytoskeletal 8 OS Homo sapiens GN KRT8 PE 1 SV 7	K2C8_HUMAN	P05787	5.34	53704	8050.384	24	14.08	**-1.9**	**-1.2**	**-2.6**	**-1.7**	-99.99	-80.60	-99.98	-90.60
211	Keratin type I cytoskeletal 14 OS Homo sapiens GN KRT14 PE 1 SV 4	K1C14_HUMAN	P02533	4.9	51904	1729.375	12	11.44	**6.8**	**-1.5**	**-2.5**	**-1.2**	99.96	-75.84	-90.78	-99.73
212	Keratin type I cytoskeletal 18 OS Homo sapiens GN KRT18 PE 1 SV 2	K1C18_HUMAN	P05783	5.17	48058	8460.187	17	12.33	**-2**	**-1**	**-1.7**	**1.2**	-100.00	-85.36	-99.94	56.40
213	Keratin type I cytoskeletal 19 OS Homo sapiens GN KRT19 PE 1 SV 4	K1C19_HUMAN	P08727	4.86	44106	12910.13	26	15.5	**-1.2**	**-1.7**	**-1.7**	**-1.5**	-98.53	-99.99	-99.88	-99.98

214	Collagen alpha 1 VIII chain OS Homo sapiens GN COL8A1 PE 1 SV 2	CO8A1_HUMAN	P27658	9.94	73478	1356.237	6	2.55	**2**	**1.2**	**2**	**2.1**	99.91	85.63	99.58	97.92
215	Collagen alpha 3 VI chain OS Homo sapiens GN COL6A3 PE 1 SV 5	CO6A3_HUMAN	P12111	6.23	345381	3354.301	59	2.93	**1.5**	**3.1**	**2.5**	**1.3**	72.87	84.82	88.47	90.78
216	Collagen alpha 1 I chain OS Homo sapiens GN COL1A1 PE 1 SV 5	CO1A1_HUMAN	P02452	5.46	139968	10166.73	43	5.4	**-1.3**	**-2.7**	**-1.7**	**6.1**	-97.72	-99.95	-99.79	100.00
217	Collagen alpha 1 VI chain OS Homo sapiens GN COL6A1 PE 1 SV 3	CO6A1_HUMAN	P12109	5.09	109670	8493.603	29	7.3	**-1.4**	**-1.5**	**-1.2**	**1.8**	-96.22	-82.01	-48.52	99.95
218	Integrin alpha V OS Homo sapiens GN ITGAV PE 1 SV 2	ITAV_HUMAN	P06756	5.31	117122	7715.855	34	5.92	**1.8**	**-1.4**	**1.1**	**4.7**	99.96	-99.26	62.49	100.00
219	Alpha internexin OS Homo sapiens GN INA PE 1 SV 2	AINX_HUMAN	Q16352	5.17	55562	1815.071	10	6.61	**-2.6**	**-3.8**	**-1.8**	**1.4**	-99.97	-99.99	-99.93	64.19
220	Interferon induced transmembrane protein 3 OS Homo sapiens GN IFITM3 PE 1 SV 2	IFM3_HUMAN	Q01628	6.59	14803	22617.93	4	21.8	**1.5**	**2.8**	**14.9**	**1.6**	75.08	99.90	98.36	68.66
221	Matrix metalloproteinase 14 OS Homo sapiens GN MMP14 PE 1 SV 3	MMP14_HUMAN	P50281	7.68	66236	2536.488	7	4.47	**1.8**	**-3.3**	**1.2**	**-1.9**	99.75	-99.71	81.09	-99.31
222	Chloride intracellular channel protein 4 OS Homo sapiens GNCLIC4 PE 1 SV 1	CLIC4_HUMAN	Q9Y696	5.29	29000	15096.56	16	15.42	**1.9**	**1.5**	**1.9**	**1**	99.81	97.38	99.96	20.50
223	ATP synthase subunit f mitochondrial OS Homo sapiens GN ATP5JK2 PE 2 SV 3	ATPK_HUMAN	P56134	9.94	11032	18487.13	3	38.3	**1.7**	**2.4**	**2.4**	**1.8**	92.19	97.26	96.90	98.82
224	ATP synthase subunit gamma mitochondrial OS Homo sapiens GN ATP5C1 PE 1 SV 1	ATPG_HUMAN	P36542	9.56	33053	5439.886	11	4.36	**1.4**	**1.2**	**1.4**	**1.8**	99.90	96.94	98.63	99.83
225	ATP synthase subunit beta mitochondrial OS Homo sapiens GN ATP5B PE 1 SV 3	ATPB_HUMAN	P06576	5.1	56560	38909.08	27	15.31	**1.3**	**1.4**	**1.5**	**1.6**	99.86	99.70	99.75	99.68
226	Perilipin 3 OS Homo sapiens GN PLIN3 PE 1 SV 3	PLIN3_HUMAN	O60664	5.14	47246	7278.349	21	15.67	**-2.1**	**-1.6**	**-1.5**	**-1.5**	-99.93	-99.80	-99.70	-99.77

227	Protein disulfide isomerase OS Homo sapiens GN P4HB PE 1 SV 3	PDIA1_HUMAN	P07237	4.56	57516	20127.57	29	2.76	**-1.4**	**-1.1**	**-1.4**	**1.1**	-99.19	-75.07	-99.76	65.82
228	Insulin like growth factor 2 mRNA binding protein 3 OS Homo sapiens GN IGF2BP3 PE 1 SV 2	IF2B3_HUMAN	O00425	9.19	64047	1668.361	14	3.45	**-1.1**	**1.2**	**-1.1**	**1**	-89.55	92.54	-47.54	28.77
229	Eukaryotic translation initiation factor 3 subunt D OS Homo sapiens GN EIF3D PE 1 SV 1	EIF3D_HUMAN	Q 15371	5.68	64600	1294.586	7	10.4	**-1.2**	**-1.1**	**-3.3**	**-1.5**	-47.18	99.52	-99.16	-84.47
230	Desmin OS Homo sapiens GN DES PE 1 SV 3	DESM_HUMAN	P17661	5.03	53593	5545.81	13	5.53	**3.3**	**1.9**	**2.6**	**1.4**	99.30	95.84	92.37	84.06
231	cAMP dependent protein kinase type II alpha regulatory subunit OS Homo sapiens GN PRKAR2A PE 1 SV 2	KAP2_HUMAN	P13861	4.77	45861	1911.196	10	12.62	**1.1**	**1.5**	**-1.8**	**-1.4**	28.63	92.82	-89.55	-94.58
232	Cofilin 1 OS Homo sapiens GN CFL1 PE 1 SV 3	COF1_HUMAN	P23528	8.22	18731	66929.81	15	33.13	**-1.5**	**-1.4**	**-1.3**	**-1.1**	-98.90	-96.24	-97.03	-78.37
233	Caveolin 1 OS Homo sapiens GN CAV1 PE 1 SV 4	CAV1_HUMAN	Q03135	5.59	20643	3448.942	6	22.47	**1.5**	**2.1**	**1.4**	**1.1**	99.91	99.63	99.95	50.07
234	Creatine kinase B type OS Homo sapiens GN CKB PE 1 SV 1	KCRB_HUMAN	P12277	5.22	42930	5774.735	7	7.35	**1.1**	**8.6**	**1.6**	**-**	74.43	100.00	99.67	-99.95
235	Caldesmon OS Homo sapiens GN CALD1 PE 1 SV 3	CALD1_HUMAN	Q05682	5.44	93288	15523.95	23	4.29	**1.2**	**-1.1**	**-1.6**	**1.3**	88.84	-92.16	-99.47	99.54
236	Galectin 1OS Homo sapiens GN LGALS1 PE 1 SV 2	LEG1_HUMAN	P09382	5.14	15058	46494.94	8	19.6	**-1.3**	**-2.4**	**-1.1**	**1.2**	-99.43	-100.00	-66.23	99.48
237	Transgelin 3 OS Homo sapiens GN TAGLN3 PE 1 SV 2	TAGL3_HUMAN	Q9UI15	7.17	22644	9376.353	9	7.04	**2**	**3.9**	**1.2**	**1.1**	99.68	99.98	76.44	45.93
238	Transgelin 2 OS Homo sapiens GN TAGLN2 PE 1 SV 3	TAGL2_HUMAN	P37802	8.45	22563	78708.73	21	14.57	**1.2**	**-1.2**	**1.2**	**-1.7**	96.64	-88.83	93.69	-99.79
239	Prohibitin 2 OS Homo sapiens GN PHB PE 1 SV 2	PHB2_HUMAN	Q99623	10.9	33296	2607.423	12	22.07	**1.2**	**1.4**	**1.3**	**-1.2**	91.52	97.26	97.49	-99.96
240	Gelsolin OS Homo sapiens GN GSN PE 1 SV 1	GELS_HUMAN	P06396	5.84	86097	16413.49	28	2.56	**-1.1**	**1.7**	**1.8**	**2.6**	-82.49	98.96	99.95	99.99

241	Thioredoxin OS Homo sapiens GN TXN PE 1 SV3	THIO_HUMAN	P10599	4.62	12022	2920.735	2	17.4	**-1**	**-2**	**-1.4**	**1.4**	-3.76	-100.00	-99.92	17.68
242	Proliferation associated protein 2G4 OS Homo sapiens GN PA2G4 PE 1 SV 3	PA2G4_HUMAN	Q9uQ80	6.11	44129	4896.417	16	17.01	**-1.5**	**-1.1**	**-1.7**	**-1.5**	-98.65	-82.93	-99.75	-99.52
243	Protein enabled homolog OS Homo sapiens Gn ENAH PE 1 SV 2	ENAH_HUMAN	Q8N8S7	6.54	66624	1701.19	6	3.21	**-1.1**	**1.4**	**-1.4**	**-1.2**	-47.18	99.52	-99.16	-84.47
244	Reticulocalbin 3 OS Homo sapiens GN RCN3 PE 1 SV 1	RCN3_HUMAN	Q96D15	4.56	37493	7251.084	15	7.32	**-2**	**-1.4**	**1.9**	**1.2**	-97.47	-97.98	99.97	98.48
245	Tubulin beta 3 chain OS Homo sapiens GN TUBB3 PE 1 SV 2	TBB3_HUMAN	Q13509	4.64	50889	51656.5	26	12.89	**-1.1**	**-1**	**1.1**	**1.2**	-92.37	-33.07	68.12	91.11
246	Rho GDP dissociation inhibitor 1 OS Homo sapiens GN ARHGDIA PE 1 SV 3	GDIR1_HUMAN	P52565	4.82	23264	12686.05	8	7.35	**1.1**	**1.1**	**1.6**	**1.2**	26.31	52.76	96.91	84.68
247	Protein S100 A4 OS Homo sapiens GN S100A4 PE 1 SV 1	S10A4_HUMAN	P26447	5.77	11957	11353.28	6	33.66	**2.8**	**-**	**12.9**	**2.2**	99.09	-62.61	99.98	99.96
248	Protein S100 A6 OS Homo sapiens GN S100A6 PE 1 SV 1	S10A6_HUMAN	P06703	5.17	10237	25335.51	7	40	**2.8**	**1**	**3.1**	**2.5**	100.00	41.38	99.99	100.00
249	Protein S100 A13 OS Homo sapiens GN S100A13 PE 1 SV 1	S10AD_HUMAN	Q99584	5.83	11471	8423.788	9	39.8	**-1.4**	**-2.9**	**1.3**	**1.4**	-99.36	-100.00	99.38	99.78
250	Protein S100 A16 OS Homo sapiens GN S100A16 PE 1 SV 1	S10AG_HUMAN	Q96FQ6	6.35	11858	5796.079	5	52.43	**-1.2**	**-1.8**	**1.6**	**-1.1**	-83.46	-99.90	99.89	-35.32
